# An advanced approach combining solid-state NMR with powder diffraction applied to newly synthesized iso­thio­uronium salts

**DOI:** 10.1107/S1600576724012378

**Published:** 2025-02-11

**Authors:** Jan Rohlíček, Václav Eigner, Jiří Czernek, Jiří Brus

**Affiliations:** a Department of Structure Analysis Institute of Physics of the Czech Academy of Sciences Na Slovance 2 Prague 18221 Czechia; b Department of Solid State Chemistry University of Chemistry and Technology, Prague Technicka 5 Prague 6 Prague16628 Czechia; c Institute of Macromolecular Chemistry of the Czech Academy of Sciences Heyrovskeho nam. 2 Prague 6 Prague16206 Czechia; Shiv Nadar Institution of Eminence, India

**Keywords:** NMR crystallograpy, powder diffraction, structure determination, iso­thio­uronium salts

## Abstract

The focus here is on the structural study of iso­thio­uronium salts and the application of intermolecular distances obtained by solid-state NMR (ssNMR) in determining crystal structures from powder diffraction data. The synthesis of three new tetra­fluoro­borate salts and two bromide salts of iso­thio­uronium compounds is presented first, followed by structural and spectroscopic studies. The tetra­fluoro­borates were further analysed using advanced ssNMR techniques to obtain a set of intermolecular ^19^F⋯^13^C, ^11^B⋯^11^B, ^1^H⋯^1^H and ^13^C⋯^1^H distances with an estimation of their precision. These distances were subsequently used as restraints in the crystal structure determination process from simulated powder diffraction data. The results show that using intermolecular distances obtained by ssNMR can increase the probability of finding the correct solution, creating new opportunities for the structural analysis of poorly diffracting compounds. This approach paves the way for solving more complex substances, such as solvates, cocrystals or complex polymorphs with many independent molecules, where traditional powder X-ray diffraction methods often reach their limits.

## Abstract 2

The synthesis of new tetra­fluoro­borate and bromide salts of iso­thio­uronium compounds is presented, followed by structural and spectroscopic studies, demonstrating that using solid-state NMR-derived intermolecular distances as a restriction can increase the likelihood of finding the crystal structure model when solving a crystal structure from powder diffraction data.

## 1. Introduction

Research focused on the synthesis and study of new compounds is inherently tied to thorough analysis, as understanding their properties and behaviours is critical. The ability to study these compounds is a crucial aspect of the process, with structural analysis being an essential part of the research. In many cases, single-crystal X-ray or electron diffraction with a precise atomic resolution is the primary method of choice. However, not all compounds are suitable for this analysis, which then requires the use of other structural techniques such as powder diffraction (PD) and solid-state NMR (ssNMR).

In the case of PD, several approaches exist to find a structural model. In addition to reciprocal-space and dual-space methods (Giacovazzo, 1998 ▸; Palatinus, 2013 ▸; Altomare *et al.*, 2009 ▸; Baerlocher *et al.*, 2007 ▸), which are commonly used in single-crystal diffraction, direct-space (DS) methods are widely used in PD to determine the structural model by applying global optimization principles. This is achieved by adjusting the position and conformation of molecular fragments within the asymmetric unit cell. Individual implementations of DS methods allow the definition of geometric constraints to facilitate the search for solutions and reduce the computational time (David & Shankland, 2008 ▸; David *et al.*, 2006 ▸; Favre-Nicolin & Černý, 2002 ▸). Continued advances in the methodology of PD, coupled with programs using different approaches to determine crystal structures from PD data, have led to the structure determination procedure becoming applicable to relatively complex crystal structures (Hušák *et al.*, 2018 ▸, 2019 ▸; Fernandes *et al.*, 2007 ▸). The recent development in DS methods has led to the speeding up of the structure determination process using the abilities of GPUs (Spillman & Shankland, 2021 ▸), reducing the degrees of freedom of the model by applying torsion angle restrictions (Kabova *et al.*, 2017 ▸), and combining PD with various techniques such as NMR, density functional theory (DFT) or theoretical prediction of the crystal structure (Habermehl *et al.*, 2022 ▸).

Despite continuous advancements, the current limitation on the complexity of DS methods, quantified by the number of degrees of freedom (DOF – encompassing free torsional angles, as well as rotational and positional coordinates), remains at approximately 40 (Hušák *et al.*, 2018 ▸, 2019 ▸; Fernandes *et al.*, 2007 ▸). Additionally, the efficacy of this methodology is significantly constrained by the quality of the diffraction pattern. Beyond instrumental influences, the primary challenge lies in the sample quality. For example, the sizes of crystalline domains and the presence of strain broaden the diffraction profile and reduce the resolution of the data, causing severe problems in solving the structure of even simple compounds (Schlesinger *et al.*, 2022 ▸).

In the case of ssNMR spectroscopy, crystal structure determination directly from experimental data is also possible. This methodology is known as NMR crystallography (Hodgkinson, 2020 ▸) and is generally based on the ability of advanced ssNMR techniques to measure intra- and intermolecular distances by analysing dipolar interactions. One of the original approaches to NMR crystallography, which allowed crystal structure determination solely from NMR data, was based on the analysis of ^1^H–^1^H spin diffusion correlation signals combined with Monte Carlo crystal structure simulations (Elena *et al.*, 2006 ▸). However, this approach requires extensive measurements of large sets of high-resolution 2D ^1^H–^1^H correlation spectra and the complex analysis of the resulting spin-diffusion build-up curves. Consequently, this methodology is not often applied. A much more promising approach, developed later, is based on the experimental determination of isotropic ^1^H and ^13^C chemical shifts and their systematic comparison with theoretical values calculated by DFT for the representative (large) set of model structures derived by the crystal structure prediction method (Salager *et al.*, 2010 ▸). The potential of this approach has recently been demonstrated on several systems with one molecule in the asymmetric part of the unit cell (Baias *et al.*, 2013 ▸; Brus *et al.*, 2016 ▸, 2018 ▸). However, the reasonable prediction of structural models of multicomponent solids such as cocrystals or polymorphic forms containing more symmetry-independent molecules requires the knowledge of key structural parameters, such as the mutual orientation of individual molecules and specific distances between them. For typical organic compounds, such information can be derived from the analysis of ^1^H–^1^H double quantum coherences (DQCs) and ^1^H–^13^C heteronuclear correlations (HETCOR) (Brown, 2012 ▸; van Rossum *et al.*, 1997 ▸; Hušák *et al.*, 2019 ▸; Brus *et al.*, 2022 ▸). For compounds containing other NMR-active nuclei, measurements of internuclear distances involving nuclei with high natural abundance and high gyromagnetic ratio, such as ^19^F, ^11^B, ^23^Na or ^31^P, are particularly convenient.

Diffraction and ssNMR structural methods are frequently employed in a complementary manner to enhance and validate each other’s findings. One of the typical bottlenecks of X-ray diffraction techniques is the determination of the hydrogen-atom position. In this case, ssNMR has allowed the identification of salts, cocrystals or tautomeric forms of compounds (Gumbert *et al.*, 2016 ▸; Smalley *et al.*, 2022 ▸). Combining ssNMR with powder diffraction is particularly advantageous as the two methods require powder samples of similar quality. An important role of ssNMR is also in the validation of the results found by powder diffraction. To study the synergy of NMR crystallography with powder diffraction in more detail, we refer the reader to the article by Harris (2022 ▸), which briefly describes the synergy of NMR spectroscopy and X-ray PD.

In the present article, we describe the preparation of iso­thio­uronium salts in the form of bromides and tetra­fluoro­borates using anion exchange. Iso­thio­uronium salts are a versatile group of compounds produced by S-alkyl­ation of thio­urea (Speziale, 1950 ▸). The variability in the thio­urea derivatives and alkyl­ating agents used results in tunability of the properties of the resulting salts. For this reason, they have found applications in numerous fields of chemistry. In organic chemistry, they are often used in the preparation of many groups of compounds such as thiols, sulfides, S-glycosides, seleno­glycourils and cytotoxic 4-amino-5-cyano-2-sulfonyl­pyrimidines (Chauhan *et al.*, 2015 ▸; Magné & Ball, 2019 ▸; Wu *et al.*, 2016 ▸; Galochkin *et al.*, 2023 ▸; Khochenkov *et al.*, 2020 ▸). They have also found application in the formation of bactericidal (Cohen *et al.*, 2017 ▸) and anticandidal (El-Zahed *et al.*, 2023 ▸) polymers as well as bactericidal micelle-forming surfactants (Valeeva *et al.*, 2021 ▸). Their antitumour activity against leukaemia cells is particularly interesting, with a selectivity index higher than 20 (Ferreira *et al.*, 2017 ▸). The cause of cell death was found to be decreased levels of anti-apoptotic protein, causing DNA damage and mitotic arrest (Assunção *et al.*, 2019 ▸). Further studies also described activities against breast (Munaretto *et al.*, 2020 ▸), melanoma (Alcolea *et al.*, 2019 ▸), lung and prostate cancer cell lines (Alcolea *et al.*, 2016 ▸). Currently, 130 crystal structures of iso­thio­uronium salts are documented in the Cambridge Structural Database (CSD; Groom *et al.*, 2016 ▸).

**Figure 1:**
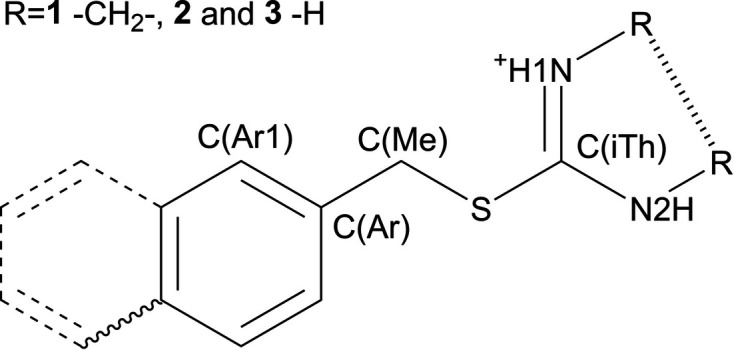
The iso­thio­uronium cation labelling scheme. The ethyl­ene bridging in **1** is depicted with a hashed bond, the methyl group in **2** is depicted with a wavy bond and the expansion to naphthyl in **3** is depicted with dashed bonds.

We synthesized 2-(benzyl­thio)-4,5-di­hydro-1*H*-imidazol-3-ium bromide (**1·Br**), 2-(benzyl­thio)-4,5-di­hydro-1*H*-imidazol-3-ium tetra­fluoro­borate (**1·BF4**), 2-(4-methyl­benzyl)­iso­thio­uronium bromide (**2·Br**), 2-(4-methyl­benzyl)­iso­thio­uronium tetra­fluoro­borate (**2·BF4**), 2-(naphthalen-2-yl­methyl)­iso­thio­uronium bromide (**3·Br**) and 2-(naphthalen-2-yl­methyl)­iso­thio­uronium bromide (**3·BF4**) and structurally describe them here, with the exception of the already published **3·Br** (Eigner, 2020 ▸). Fig. 1 ▸ shows the molecular scheme.

This work presents a comprehensive structural and spectroscopic study of these newly synthesized iso­thio­uronium salts. We utilized their structural models to evaluate a novel combined approach employing ssNMR and PD for crystal structure determination. These compounds are relatively simple in terms of DOF and diffract very well, making it straightforward to determine their crystal structures by powder diffraction. Therefore, to test the abilities of the new approach, we applied it to calculated data with significant peak broadening to simulate nanocrystalline or strained samples, which makes the structure solution problematic. Specific ssNMR experiments were conducted to analyse ^19^F–^13^C, ^11^B–^11^B, ^1^H–^1^H and ^1^H–^13^C correlations, allowing us to estimate the corresponding intermolecular distances. These distances were then used as additional restraints in the crystal structure determination process to assess the efficacy of this new methodology.

## 2. Results and discussion

### 2.1. Synthesis of iso­thio­uronium compounds

All the materials used in the preparation of the iso­thio­uronium salts were purchased from commercial suppliers (Merck, TCI, Penta) and used without further purification.

Bromides **1·Br**, **2·Br** and **3·Br** were prepared using an equimolar ratio of thio­urea (2-imidazoline­thione) and aryl­bromide. The thio­urea was dissolved (suspended) in aceto­nitrile (20 ml) and to the resulting solution (suspension) the corresponding amount of aryl­bromide was added. The reaction mixture was stirred using a magnetic stirrer at room temperature for 3 h. The resulting precipitate was filtered off and dried.

Tetra­fluoro­borates **1·BF4**, **2·BF4** and **3·BF4** were prepared from the bromides using anion exchange. The iso­thio­uronium bromides were suspended in distilled water (3 ml) with an equimolar amount of sodium tetra­fluoro­borate. The reaction mixture was shaken at 350 rpm at room temperature for one week. The resulting solid was then filtered off, washed with distilled water (3 ml) and allowed to dry. The resulting material was crushed in an agate mortar with a pestle and shaken in distilled water at 350 rpm at room temperature for a week to dissolve any remaining inorganic salts. The material was then filtered off and allowed to dry. The procedure was unsuccessful for samples **1·BF4** and **2·BF4**. These samples were then subjected to the same treatment a second time, but replacing the equimolar amount of sodium tetra­fluoro­borate and distilled water with a saturated solution of sodium tetra­fluoro­borate (3 ml). The transition under these conditions was successful.

### 2.2. Liquid NMR and IR spectroscopy

The prepared compounds were analysed using ^1^H NMR and ^13^C NMR in perdeuterated dimethyl sulfoxide. The NMR analysis confirmed the structures of the prepared compounds, and in the cases of **1·Br**, **1·BF4**, **2·Br** and **2·BF4** the spectra did not show any significant differences before and after the anion exchange. However, in the case of **3·Br**, splitting of iso­thio­uronium NH_2_ peaks was observed. After the anion exchange to **3·BF4** the NH_2_ peaks merged, forming a single broad peak. For detailed IR and NMR results see Section S1 and Fig. S1 in the supporting information.

### 2.3. Crystallographic study

**Figure 2:**
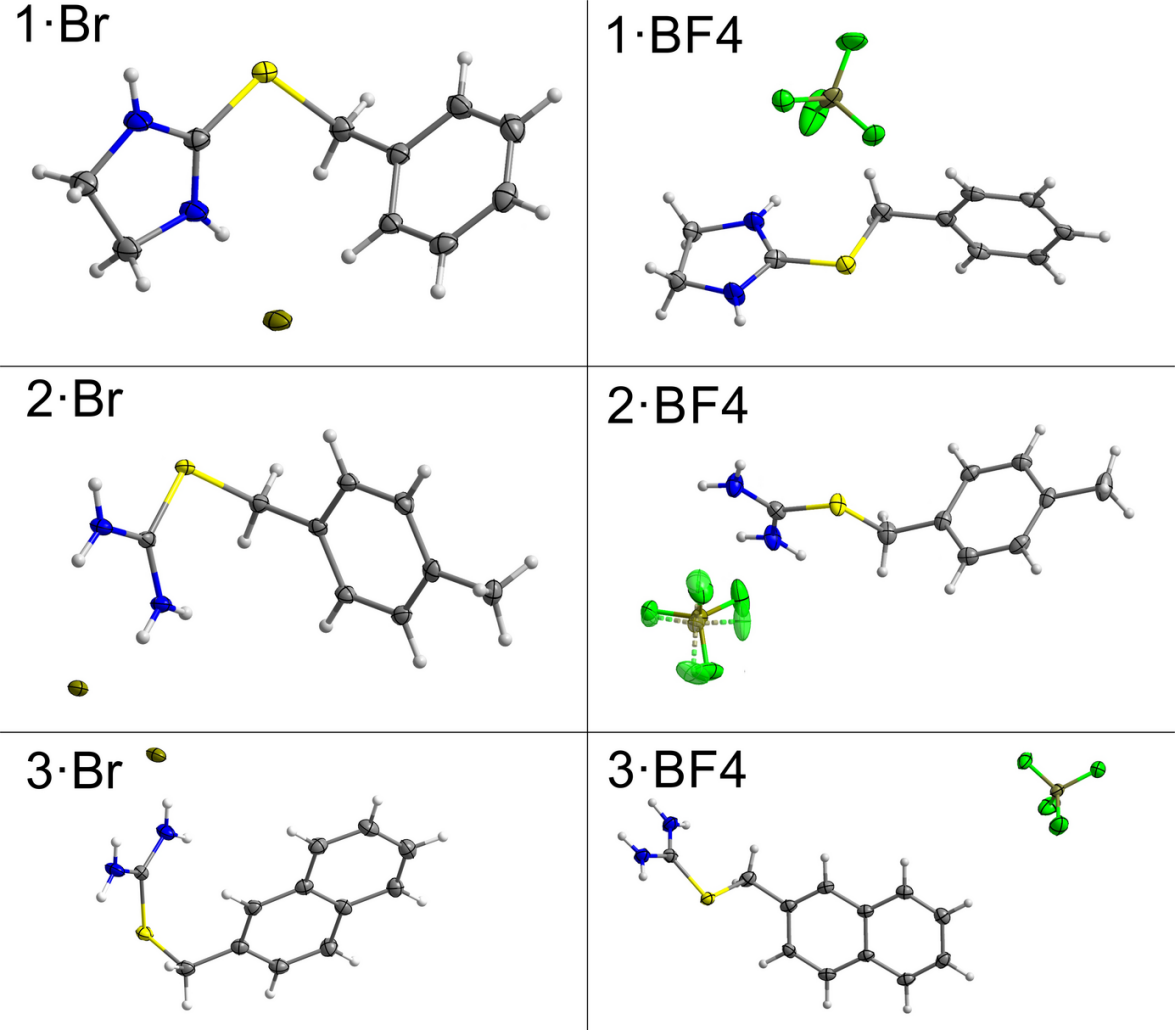
The asymmetric parts of the unit cells of the studied compounds, with displacement ellipsoids drawn at the 50% probability level. Weakly occupied atoms are depicted as transparent with dashed bonds.

All presented structures crystallized in the monoclinic system: samples **1·Br**, **1·BF4** and **2·Br** in centrosymmetric *P*2_1_/*c* and *P*2_1_/*n* space groups, and **2·BF4** and **3·BF4** in the non-centrosymmetric *P*2_1_ space group (Table 1 ▸). In all cases, the asymmetric unit consisted of one iso­thio­uronium cation and one anion, which is disordered over two positions in **2·BF4** (Fig. 2 ▸). The published structure **3·Br** (Eigner, 2020 ▸) is included in the discussion for completeness. Due to the differences in atomic labelling, we have assigned common labels for all the cations that will be used for the description of structural differences in this work (Fig. 1 ▸). The bond lengths and angles vary little among the studied compounds and do not significantly differ from the expected values. Large differences between C(Me)—S and C(iTh)—S can be attributed to the partial double-bond character of the C(iTh)—S bond. The differences among the bond angles are more pronounced; there is a clear tendency in the bromides towards higher C(Ar)—C(Me)—S angle values, with an average value of 112.9°, while the tetra­fluoro­borates tend towards lower C(Ar)—C(Me)—S angle values, with an average value of 107.3°. Another significant difference can be observed among the S—C(iTh)—N1 and S—C(iTh)—N2 angles; the corresponding angles are more obtuse in compounds **1·Br** and **1·BF4** with average values of 127.7° and 121.1°, respectively, while for **2·Br**, **2·BF4**, **3·Br** and **3·BF4** the average values are 121.8° and 116.8°. These differences are most likely caused by the steric requirements of the five-membered ring present in structures **1·Br** and **1·BF4**. Among the newly presented crystal structures, the C(Me)—S—C(iTh) angle exhibits a small variance, with the largest deviation being 1.6° from the average value of 102.7°, while for the published structure **3·Br**, the corresponding angle has a value of 96.99 (16)°. For further information on bond lengths and angles, see Tables S1 and S2 in the supporting information.

**Figure 3:**
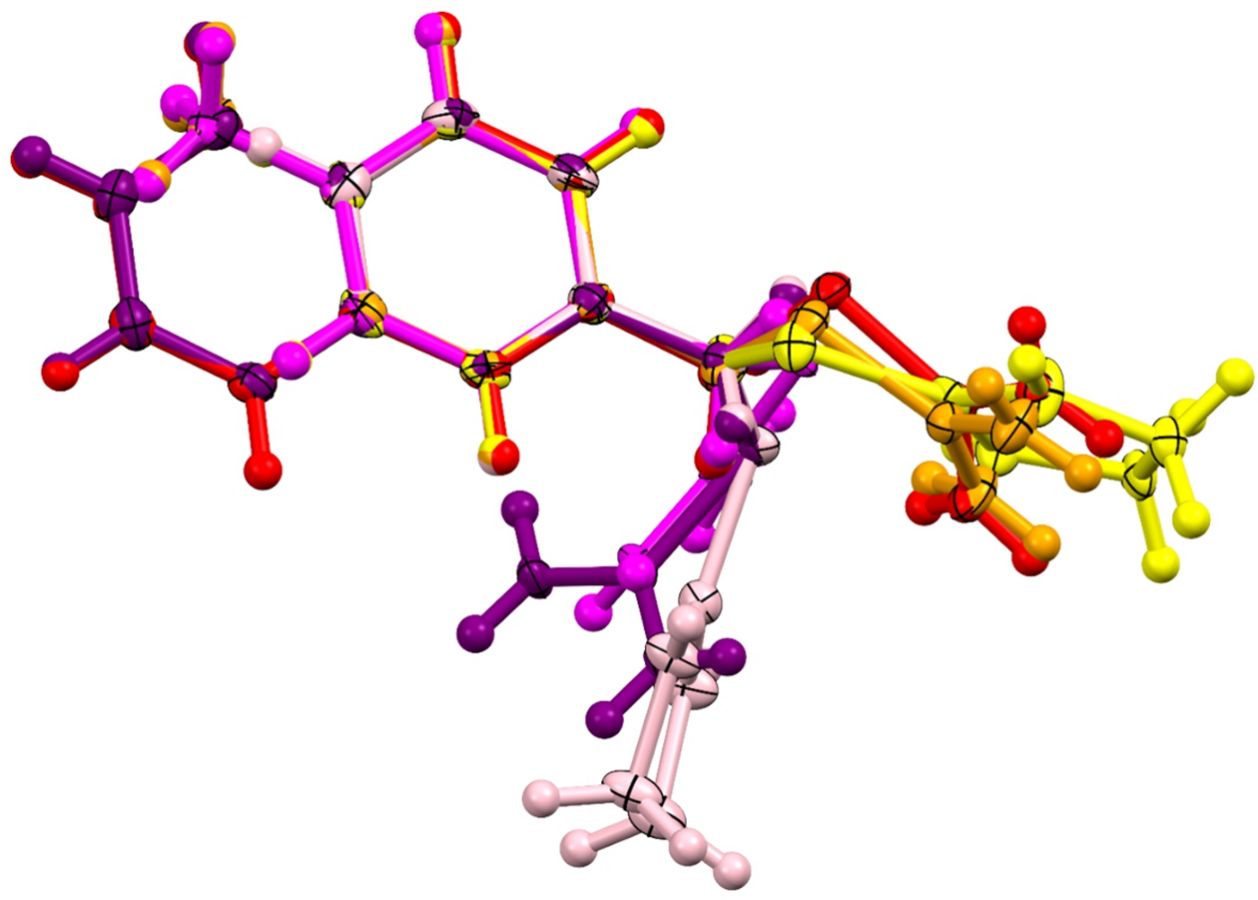
Overlay of the iso­thio­uronium cations. Cations from **1·Br**, **2·Br** and **3·Br** are depicted in pink, magenta and purple, respectively, and cations from **1·BF4**, **2·BF4** and **3·BF4** are depicted in yellow, orange and red, respectively.

The possible rotation of two single bonds, C(Ar)—C(Me) and C(Me)—S, and one partial single bond, S—C(iTh), allows for conformational changes in the structures of the studied compounds. Among the newly studied compounds, rotation about the partial single bond S—C(iTh) appears to be very constrained, with an average absolute value of the torsion angle C(Me)—S—(iTh)—N2 of 168.6° and the largest difference being 5.3° in the case of structure **1·BF4**. In structure **3·Br**, the corresponding torsion angle is 110.7 (3)°. The rotation about C(Ar)—C(Me) does not seem to follow any structure-related trend, but in all the structures it is significantly different from a planar arrangement, most likely due to steric interference with the H atoms of the aromatic ring. The average absolute value of the C(Ar1)—C(Ar)—C(Me)—S torsion angle is 101.6° with the largest difference being 26.7° in structure **2·Br**. In the case of rotation about the C(Me)—S single bond, a clear structure-related trend is observed. Among the bromides, the iso­thio­uronium cation bends significantly, with an average absolute value of C(Ar)—C(Me)—S—C(iTh) of 76.2° and the largest difference being 13.2° in the case of **1·Br**. Among the tetra­fluoro­borates, the cations are almost straight, with the average absolute value of C(Ar)—C(Me)—S—C(iTh) being 168.6° and the largest difference being 5.3° in structure **3·BF4** (Fig. 3 ▸). The straightening of the iso­thio­uronium cation in the structures of the tetra­fluoro­borates is most likely caused by the anisotropic behaviour of the tetra­fluoro­borate anion, which only allows the formation of strong hydrogen bonds in specific directions. However, the bromide anion can form hydrogen bonds in almost any direction, giving the weaker non-covalent interactions a larger influence on the cation conformation. For further instrumental and structural descriptions see Section S3 and Table S1–S5 in the supporting information.

### 2.4. ssNMR spectroscopy

Before the ssNMR analysis, the purity of the powdered samples was tested by phase analysis (Section S6 and Figs. S23–S25 in the supporting information).

**Figure 4:**
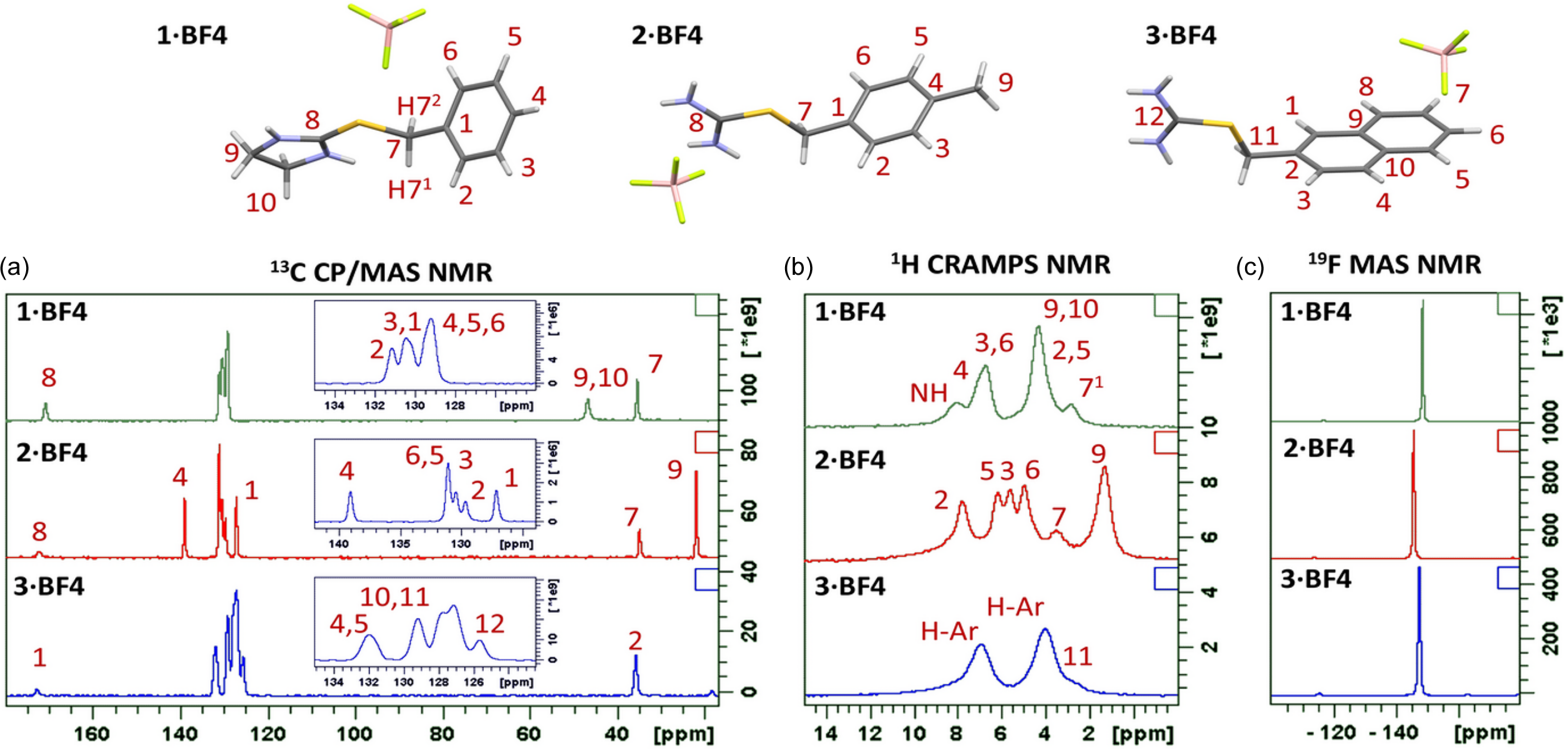
(*a*) ^13^C CP/MAS NMR, (*b*) ^1^H CRAMPS and (*c*) ^19^F MAS NMR spectra of the crystalline compounds **1·BF4**, **2·BF4** and **3·BF4**. The molecular structures with the atom numbering are displayed above the spectra. H atoms are numbered according to their parent atoms, so H2 is on C2, H3 is on C3, H7^1^ and H7^2^ are on C7, *etc.*

A prerequisite for reliable determination of interatomic distances from NMR spectra is sufficient spectral resolution to allow unambiguous identification of individual atoms. However, as the structural differences between the aromatic C atoms are relatively small, not all the signals are resolved in the ^13^C cross-polarization/magic-angle spinning (CP/MAS) NMR spectra [Fig. 4 ▸(*a*)]. This issue is much more complex for ^1^H combined rotation and multipulse spectroscopy (CRAMPS) NMR spectra [Fig. 4 ▸(*b*)], where the spectral resolution and chemical shift dispersion are strongly dependent on the structural diversity of the molecule and the presence of specific non-covalent interactions, *e.g.* hydrogen bonding. Nevertheless, by complementing the data with two-dimensional (2D) ^1^H–^13^C frequency switched Lee–Goldburg (FSLG) HETCOR, ^1^H–^1^H double-quantum/single-quantum (DQ/SQ) CRAMPS and ^19^F–^13^C CP/MAS NMR spectra and quantum chemical calculations (Brus *et al.*, 2016 ▸), all the key signals were assigned reliably (for details see Section S4, Tables S6–S8 and Figs. S9–S11).

Due to the methyl substitution, the structural differences between the aromatic H atoms are sufficient to be resolved in ^1^H CRAMPS NMR. Consequently, all ^1^H resonances can be distinguished for **2·BF4** [Fig. 4 ▸(*b*)]. For both **3·BF4** and **2·BF4**, the signals of the NH_2_ H atoms are broadened due to the strong dipolar interactions with ^14^N and due to the resonance effects involving NH and NH_2_ groups. In the absence of a methyl unit in the molecule of **1·BF4**, the ^1^H spectral resolution is again slightly reduced. Nevertheless, at least two key resonances can be used to trace inter- or intra-molecular polarization transfers. Namely, it is the resonance of the CH7^1^ H atom at 2.85 p.p.m. and the signal of NH H atoms resonating at 8.21 and 7.94 p.p.m.

When looking at the BF_4_^−^ counterion, the narrow symmetric ^11^B MAS NMR signals at *ca* −1 p.p.m. detected for all systems (Section S5, Fig. S12) indicate tetrahedral coordination of the B atom, the local geometry of which is highly symmetrical and probably effectively motion averaged due to the tumbling of the BF_4_^−^ ion. This assumption is further supported by the ^19^F MAS NMR spectra [Fig. 4 ▸(*c*)] in which single narrow signals at *ca* −145 p.p.m. are detected. This finding thus indicates the structural and magnetic equivalence of all F atoms in the BF_4_^−^ anion caused by the reorientation of BF_4_^−^ anions in the crystal structure.

#### 2.4.1. Measurement of ^19^F⋯^13^C interatomic distances

In NMR spectroscopy, information about interatomic distances *r*_IS_ is generally encoded in the strength of dipolar interactions *D*_IS_ (*D*_IS_ ≃ 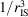). Consequently, the measurement of internuclear distances is limited to a relatively narrow range when the maximum distances that can be reliably measured do not exceed a length of about 10 Å in ideal conditions (Yuen *et al.*, 2010 ▸). This is because there are no measurable dipolar interactions between more distant spins. Owing to the absence of observable very long range dipolar interactions, the detected NMR signals do not show any additional oscillation or evolution that can be interpreted in terms of internuclear distances. In practice, however, due to experimental imperfections and other unwanted effects such as dipolar truncation (Bayro *et al.*, 2009 ▸), the typical maximum interatomic distance detected in organic solids is usually no greater than 6–8 Å.

Since there is only one type of ^19^F atom in the studied compounds, the simplest way to probe dipolar couplings between ^19^F and ^13^C heteronuclei is a variable contact time cross-polarization experiment. The strength of the dipolar interactions is then inversely proportional to the time constant *T*_IS_, which describes the initial rate of the build-up of ^13^C NMR signals as formed by the cross polarization from ^19^F spins. This polarization transfer is described by the following function:
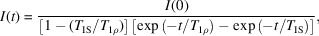
where *T*_1ρ_ describes spin–lattice relaxation in the rotating frame. Since the time constant *T*_IS_ is proportional to the third power of the interatomic distance, we first calibrated the *T*_IS_ ≃ *r*^3^ dependence using the parameters determined for the crystalline molecular system with known local geometry and derived calibration function. As the investigated systems contain the BF_4_^−^ ion, which is used as a probe for the measurement of ^19^F⋯^13^C interatomic distances, we calibrated the rate of ^19^F–^13^C polarization transfer using the model crystalline compound sodium tri­fluoro­acetate (TFA), which contains a CF_3_ unit. This CF_3_ unit is also represented by a single ^19^F MAS NMR signal, suggesting some rotational motion or jumps. Consequently, in this calibration the influence of the existence of three spectroscopically unresolved F atoms is also involved. Therefore, we believe that the TFA model system with the CF_3_ functional group is structurally close enough to the structural motifs in the investigated systems with the BF_4_^−^ anion to provide a representative model that can be used to calibrate the polarization transfer from BF_4_^−^ ions. Bearing in mind all the complexity of the cross-polarization transfer, which depends not only on interatomic distances but also on local mobility and the number of interacting spins (Kolodziejski & Klinowski, 2002 ▸), the time constants *T*_IS_ = 0.5 ± 0.1 and 1.6 ± 0.2 ms and the corresponding distances of *ca* 1.4 and 2.5 Å obtained for the model TFA system basically follow the expected dependence (see Section S5.1 and Figs. S14 and S15). This dependence was then used to convert the determined *T*_IS_ constants to ^19^F⋯^13^C interatomic distances.

**Figure 5:**
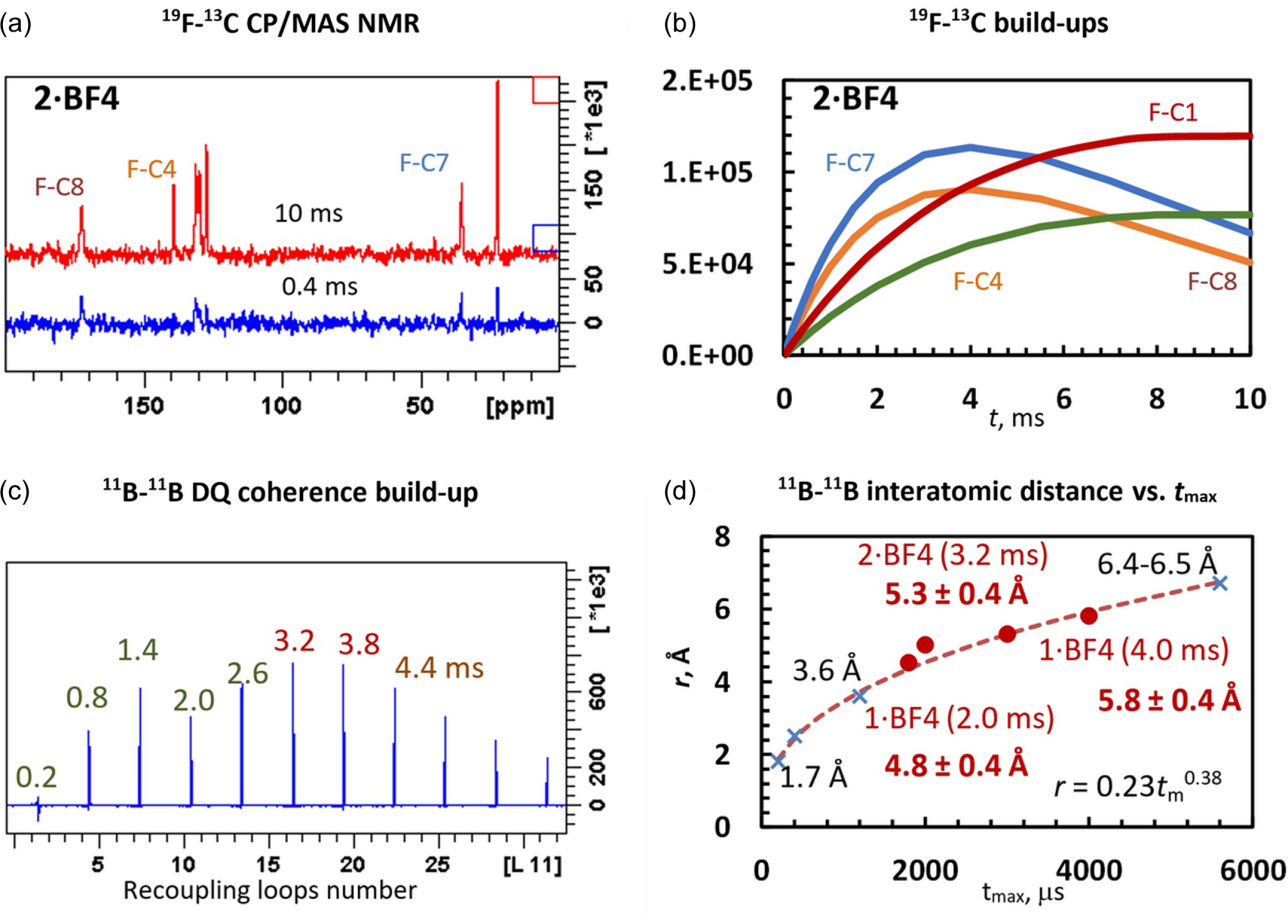
(*a*) ^13^C{^19^F} CP/MAS NMR spectra of crystalline **2·BF4** measured at two different cross-polarization mixing times. (*b*) ^19^F–^13^C cross-polarization build-up curves created for atoms C1, C4, C7 and C8. (*c*) A typical ^11^B–^11^B DQC build-up recorded for **2·BF4**. (*d*) The dependence between the recoupling time at maximum DQ coherence intensity *t*_m_ and the interatomic ^11^B⋯^11^B distance *r*. The relation *r* = 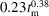, where *r* represents the ^11^B⋯^11^B interatomic distance and *t*_m_ is the recoupling time to reach maximum signal intensity, was derived previously by fitting the *t*_m_ recoupling times experimentally determined for crystalline compounds with known B⋯B interatomic dissonances such as CsCoD, borax, H_3_BO_3_ or 2-methyl­propyl­boronic acid (Brus *et al.*, 2017 ▸; Hušák *et al.*, 2018 ▸).

Fig. 5 ▸(*a*) demonstrates typical ^13^C{^19^F} CP/MAS NMR spectra measured at different ^19^F–^13^C cross-polarization mixing times (0.4 and 10 ms, **2·BF4** compound). The build-ups of the corresponding ^13^C{^19^F} CP/MAS NMR signals are then presented in Fig. 5 ▸(*b*), and the complete experimental data collected for all compounds are summarized in Section S5.2 and Fig. S16. The corresponding *T*_IS_ time constants, together with the ^19^F⋯^13^C interatomic distances estimated using the derived calibration function, are listed in Table S9. Since the time constants *T*_IS_ were determined with an experimental error of *ca* ±0.5–0.7 ms, the uncertainty in the estimated distances is at least about ±0.2 Å. However, bearing in mind other contributions affecting the determination of the *T*_IS_ constants, such as partial overlap of ^13^C resonance frequencies, local static disorder, motion averaging of dipolar couplings caused by the supposed rotation of the BF_4_^−^ anion or the number of interacting spins, we suppose that our measurement is burdened with an additional uncertainty. Consequently, we assume that the interatomic ^19^F⋯^13^C distances are rather estimated with an experimental error of about ±0.3–0.4 Å.

For the **2·BF4** compound, for instance, the fastest increase in the signal intensities was observed for atoms C7 and C8, for which the cross-polarization ^19^F–^13^C rate constants *T*_IS_ were determined to be 2.8 and 3.3 ± 0.5 ms, respectively. This indicates a shortest interatomic distance of about 3.1–3.3 ± 0.4 Å. A slightly slower signal build-up with a *T*_IS_ of 6.6 ms was observed for atom C1, which reflects a slightly more distant ^19^F–^13^C spin pair of *ca* 4.2 ± 0.4 Å. The slowest build-up characterized by the longest *T*_IS_ time constant of 8.6 ms was then detected for atom C4, reflecting an inter­atomic distance of about 4.6 ± 0.4 Å. Overall, the short-range one-bond F⋯C distances of *ca* 1.4 ± 0.2 Å are characterized by *T*_IS_ constants of about 0.5 ms, while the two-bond spin pairs of *ca* 2.5 ± 0.2 Å have *T*_IS_ constants of about 1.5 ms. The medium-range F⋯C distances up to *ca* 3.0–4.0 ± 0.4 Å are typically reflected by *T*_IS_ ranging from 2.7 to 5.0 ms, whereas the long-range distances of about 4.2–5.0 ± 0.4 Å have *T*_IS_ of about 6–10 ms.

In this context it is worth mentioning that the use of cross-polarization techniques to monitor interatomic distances requires careful Hartmann–Hahn matching to the central band condition. When the experiment is Hartmann–Hahn matched to the ±1 spinning side band condition, especially at high MAS frequencies, the ^13^C{^19^F} CP/MAS NMR signal build-up exhibits a dipolar oscillation, the precise detection of which is very time consuming (van Rossum *et al.*, 2000 ▸). When matched to the central Hartman–Hahn condition, the dipolar oscillation is suppressed and the interatomic distance can be probed via analysis of the initial build-up of the ^19^F–^13^C signals. However, since the experiment does not work with precisely defined spin pairs, and the *T*_IS_ parameter rather operates with the polarization transfer between spin *baths*, such an analysis requires calibration using standard systems. To avoid this problem there are other methods that can be used to monitor heteronuclear dipolar interactions in solids, and among them the rotational echo double resonance (REDOR) technique is one of the most efficient (Shcherbakov & Hong, 2018 ▸).

Note also that explicit signal assignment and a high level of spectral resolution, when all signals are separated, are beneficial for obtaining reliable distance information. The presence of disorder or signal overlap may reduce the accuracy of the derived structural parameters (Cordova *et al.*, 2023 ▸). However, in the systems investigated, such local disorder of the BF_4_^−^ anion had only a limited effect on the results obtained. Nevertheless, further research is needed in this direction, particularly to identify the limitations and possibilities of structure determination of more disordered and near-amorphous organic solids.

#### 2.4.2. Measurement of ^11^B⋯^11^B interatomic distances

^11^B nuclei, owing to their high gyromagnetic ratio and high natural isotopic abundance, are particularly suited to probing long-range dipolar contacts in multicomponent systems and, as demonstrated previously, the evolution of ^11^B–^11^B DQC allows the determination of ^11^B⋯^11^B distances up to *ca* 7 Å (Brus *et al.*, 2017 ▸; Hušák *et al.*, 2018 ▸). Such a typical build-up of ^11^B–^11^B DQC showing a maximum at *ca t*_m_ = 3.2 ± 0.4 ms is demonstrated in Fig. 5 ▸(*c*) for **2·BF4**. By applying the previously derived calibration function *r* = 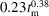, where *r* represents the ^11^B⋯^11^B interatomic distance and *t*_m_ is the recoupling time at which the DQC reaches maximum intensity [Fig. 5 ▸(*d*)], the *t*_m_ values indicate typical ^11^B⋯^11^B interatomic distances of *ca* 5.3 ± 0.4 Å. When considering the existence of a distribution of interatomic distances, for instance by the presence of two B–B pairs, then the corresponding typical interatomic distances could be *ca* 5.0 and 5.5 ± 0.4 Å. The distances obtained in this way for all the investigated compounds are summarized in Table S10. Also, in this case, the determined B⋯B distances must be considered as relatively rough approximations. This is mainly because the build-up of ^11^B NMR signals is not only driven by the evolution of the DQCs of the two interacting ^11^B spins in the spin pair but also influenced by additional relatively strong interactions with the directly coupled ^19^F spins. These interactions can cause additional oscillations of the ^11^B NMR signals and thus have an effect on the determination of the ^11^B⋯^11^B interatomic distance. However, due to hardware limitations, these ^19^F–^11^B interactions could not be eliminated.

#### 2.4.3. ^1^H–^1^H DQ/SQ CRAMPS NMR correlations

**Figure 6:**
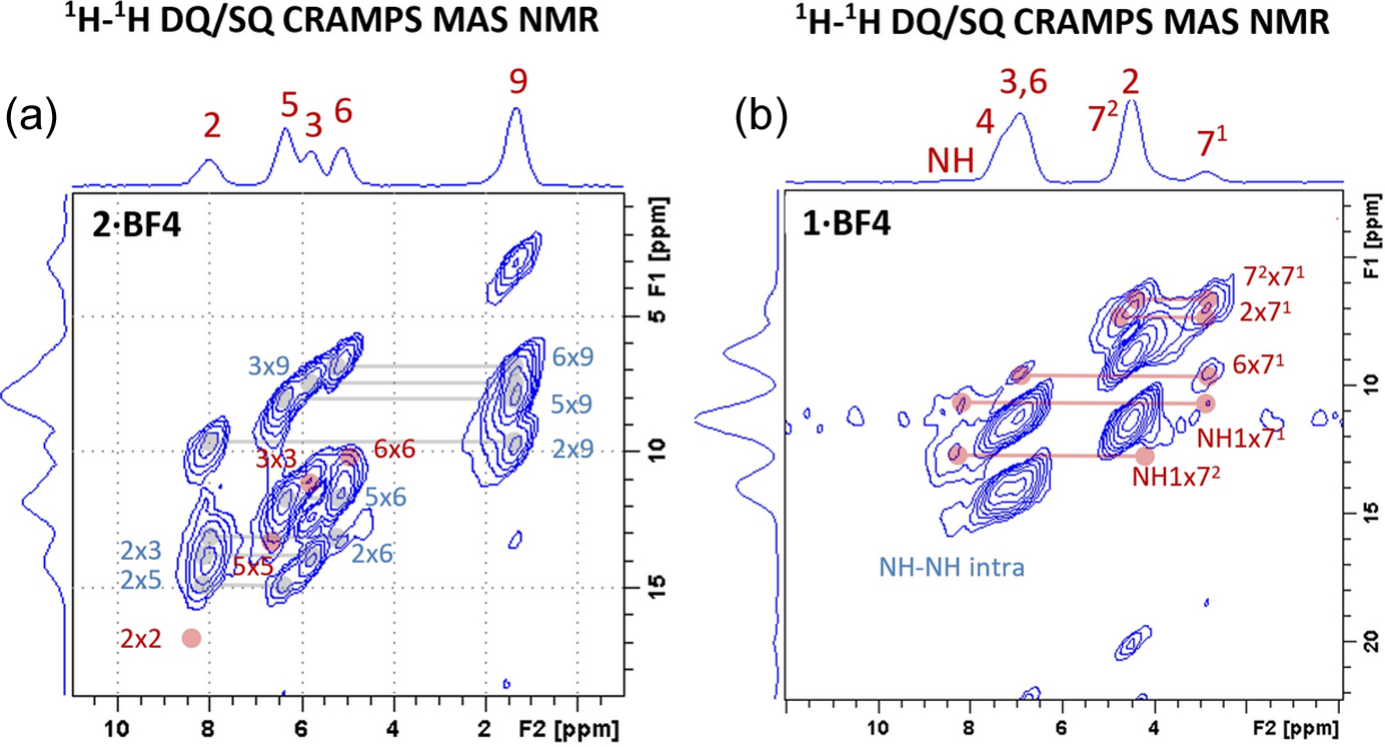
^1^H–^1^H DQ/SQ CRAMPS NMR correlation spectra for (*a*) **2·BF4** and (*b*) **1·BF4** measured at a spinning frequency of 10 kHz and with four recoupling loops. The short-range intramolecular ^1^H–^1^H correlation signals, *e.g.* between atoms H9 and H3, H6 or H5, are represented by blue labels 3×9, 6×9 or 5×9, respectively. The medium- and long-range intermolecular ^1^H–^1^H autocorrelation signals involving, for example, the aromatic atoms H2, H3, H5 and H6 are shown as red dots and labelled 2×2, 3×3, 5×5 and 6×6, respectively.

If the resolution of a ^1^H CRAMPS spectrum is good enough, then the corresponding 2D ^1^H–^1^H DQ/SQ CRAMPS NMR spectra (Fig. 6 ▸) can be used to trace ^1^H⋯^1^H interatomic dipolar contacts in order to obtain additional information on the corresponding distances. For the system **2·BF4** and due to the good resolution of ^1^H resonances, the corresponding 2D ^1^H–^1^H DQ/CQ MAS NMR spectrum [Fig. 6 ▸(*a*)] shows an almost complete set of correlation signals, reflecting dipolar contacts between ^1^H spins. However, the majority of the detected correlation signals reflect structurally nearly useless intramolecular short-range ^1^H⋯^1^H dipolar contacts, which do not provide essential information on molecular packing. Moreover, these correlation signals, *e.g.* between atoms H9 and H3, H6 or H5 [in Fig. 6 ▸(*a*) represented by blue labels 3×9, 6×9 or 5×9, respectively], remain strong even in the spectra measured with a relatively large number of recoupling loops (*L* = 4 or 5, Figs. S18 and S19). In such a case, much more useful are the ^1^H–^1^H autocorrelation signals between aromatic CH H atoms, since they only form when two mol­ecules are appropriately oriented and relatively very close to each other. As the autocorrelation signals only evolve when the corresponding atoms are sufficiently close together (no more than about 5.0–5.5 Å), their presence or absence basically defines the molecular arrangement in the crystal structure. For the **2·BF4** compound, we focused on the autocorrelation signals involving the aromatic atoms H2, H5, H3 and H6, whose signals are well separated [in Fig. 6 ▸(*a*) these autocorrelation signals are shown as red dots and labelled 2×2, 3×3, 5×5 and 6×6].

Specifically, the presence of strong H3–H3 autocorrelation signals and a slightly weaker H6–H6 autocorrelation signal indicates that the corresponding interatomic distances are less than *ca* 4.5 Å, whereas the absence of H2–H2 even recorded with the longest recoupling time indicates that the corresponding intermolecular interatomic distance must be longer than *ca* 5.0–5.5 Å (Brus *et al.*, 2016 ▸). Similarly, for the **1·BF4** compound, we focused only on the analysis of the ^1^H–^1^H correlations involving the signals of CH_2_ atom H7^1^ [Fig. 6 ▸(*b*)]. Besides the expected very short intramolecular H7^1^⋯H7^2^ pair with a distance of *ca* 1.8 Å, we identified a second short-range pair (probably intermolecular) involving atom H2. Bearing in mind the relatively high intensity of the correlation signal and relatively short recoupling times, the distance in this H7^1^⋯H2 pair can be estimated in the range of *ca* 2.0–2.5 Å. All the recorded spectra and extracted distances are summarized in Figs. S17–S19 and Table S11.

#### 2.4.4. ^1^H–^13^C HETCOR MAS NMR correlation

**Figure 7:**
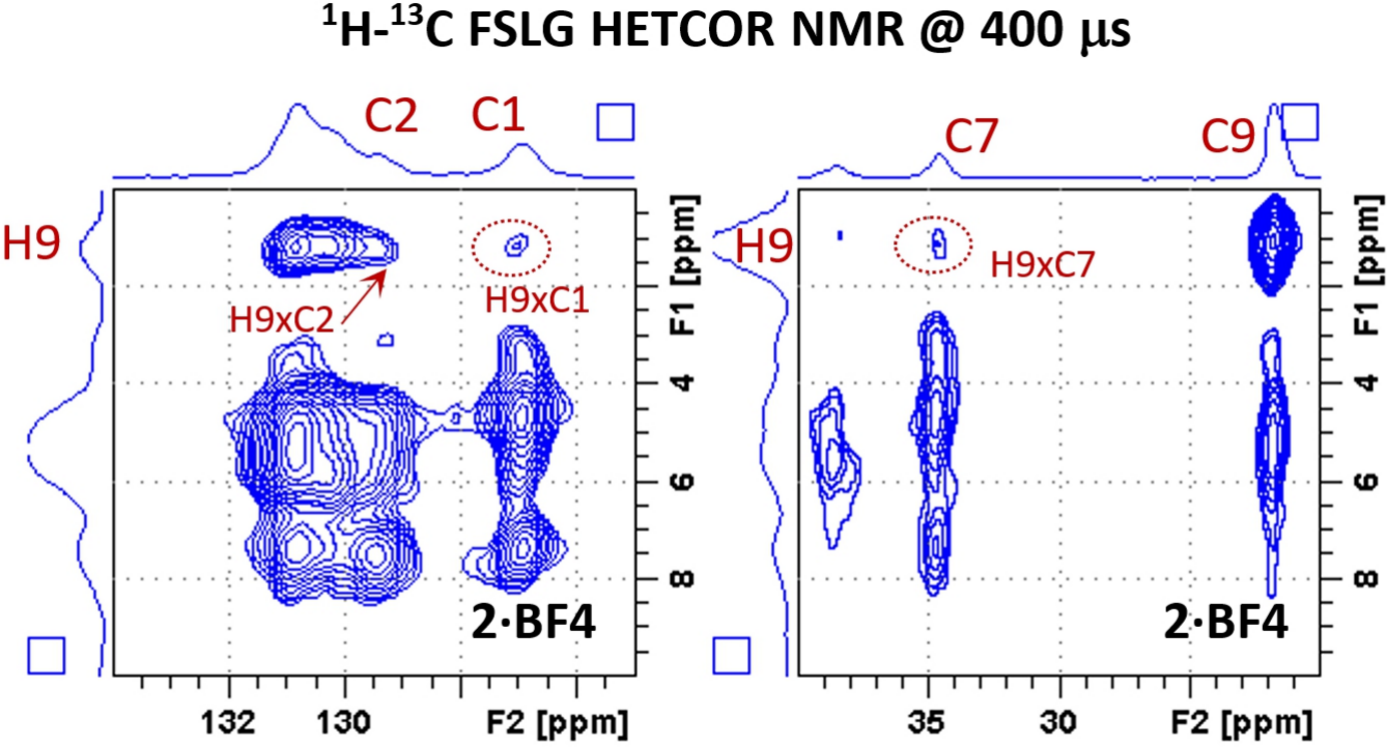
Expanded regions of the ^1^H–^13^C FSLG HETCOR NMR spectrum of the **2·BF4** compound measured at 400 µs cross-polarization contact time.

On the same lines as for the ^1^H–^1^H correlation experiments, useful information about the interatomic distances between ^1^H and ^13^C nuclei could be derived from the ^1^H–^13^C HETCOR MAS NMR spectra, but only for the **2·BF4** system, which had a very good resolution in the ^1^H dimension (Fig. 7 ▸). Specifically, we monitored the ^1^H polarization transfer from methyl H atoms (H9). This is because the ^1^H resonances of methyl groups usually exhibit long lifetimes, allowing large distances to be bridged. In addition, the intramolecular distances between atoms H9 and C7 and C1 of about 6.2 and 4.7 Å, respectively, are too large to allow efficient polarization transfer. Consequently, the H9–C7 correlation signal detected within the 400 µs CP mixing time (Fig. 7 ▸ and Fig. S20) must reflect intermolecular contacts. Following the literature data (van Rossum *et al.*, 1997 ▸; Brus & Jegorov, 2004 ▸), the corresponding interatomic distances are about 3.5–4.0 Å, because the intramolecular H9–C2 correlation signal reflecting a similar distance is of a comparable intensity. In this regard, the correlation signal H9–C1 then seems to indicate an intermolecular dipolar contact reflecting a similar interatomic distance (<4.0 Å), because the corresponding intramolecular distance is considerably larger (4.7 Å).

In summary, by using a range of experimental techniques, we have obtained a representative set of intermolecular distance restraint data involving ^19^F⋯^13^C pairs (12×), ^11^B⋯^11^B pairs (3×), ^1^H⋯^1^H pairs (7×) and ^13^C⋯^1^H pairs (2×) which were subsequently used for crystal structure determination from simulated X-ray PD data (Table 2 ▸ and Table S12).

### 2.5. Applying and testing the combined approach of ssNMR and PD

We modified the source code of the *FOX* program (Favre-Nicolin & Černý, 2002 ▸) to apply the distances obtained by ssNMR to the structure determination process from PD data. The global optimization algorithm in *FOX* uses a cost function (CF) to evaluate the quality of the model and searches for a solution by minimizing the CF. The CF includes the quality of the profile fit and several other terms that reflect the additional restraints applied. The equation of the cost function used in *FOX* can be written as
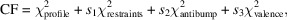
where individual χ^2^ are terms for agreement of the profile fit, geometric restraints, anti-bump and bond valences, and *s_i_* are their scale factors. We took advantage of this definition and introduced an additional parameter reflecting the agreement of the intermolecular distances (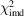), defined in the same way as 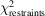 (Favre-Nicolin & Černý, 2004 ▸), and its scale factor *s*_4_,
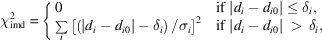
where *d_i_* is the actual value, *d*_*i*0_ is the defined restraint value, δ_*i*_ means the range without penalty, σ_*i*_ plays the role of the precision of the defined value and imd (or IMDs) abbreviates the term intermolecular distances.

The intermolecular distances obtained by ssNMR, summarized in Table 2 ▸, were used in the crystal structure determination process of compounds **1·BF4**, **2·BF4** and **3·BF4** from simulated X-ray PD data. These compounds are relatively simple to solve because they have only 15 degrees of freedom. This also confirmed the initial testing with simulated data corresponding to the laboratory instrument (FWHM = 0.1° 2θ), where almost all runs executed with 10^6^ trials ended up with a correct solution. With such a resolution and simple compounds, the structure solution process from powder data is straightforward and the impact of additional information on the success rate is negligible. Additional information in the structure determination process is useful only in situations where the success rate is small or even zero. To create a scenario where finding the structure solution is challenging, we simulated low-quality data. We generated two theoretical X-ray PD patterns for each of **1·BF4**, **2·BF4** and **3·BF4** in the program *Mercury* (Macrae *et al.*, 2020 ▸) (from 4° to 50° 2θ, step size 0.01, λ = Cu *K*α_1_) with significant peak broadening. We set the FWHMs to 0.5° and 1.5° 2θ to simulate bad and extremely bad diffraction data, respectively. These simple simulations would be more appropriate for a situation where too wide a slit has been used than for badly diffracting samples, where the peak broadening is usually induced by stressed crystallites, small particles or a combination of the two, and the profile is difficult to describe with the available profile functions.

**Figure 8:**
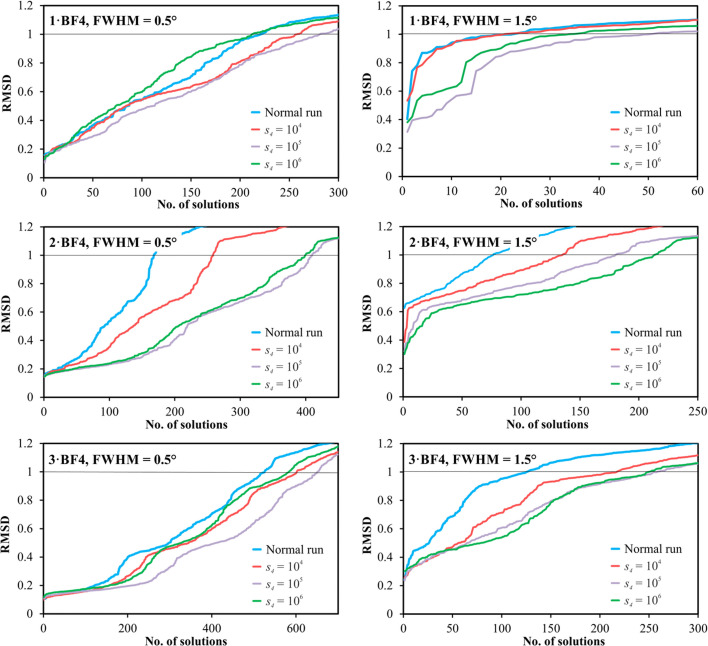
Individual graphs showing a sorted list of solutions by r.m.s.d. (only the *N* best solutions out of 1000 are depicted for clarity in each graph) of the structure determination process of **1·BF4**, **2·BF4** and **3·BF4** from simulated X-ray PD data and their similarity to the reference crystal structures as an r.m.s.d. value of the closest atomic positions in the overlapped molecular clusters. Patterns with FWHM = 0.5° (left) and FWHM = 1.5° (right) were used. Approximately at r.m.s.d. = 1 Å, the solutions lost their similarity to the reference structure. The black line in all graphs notes this value. Individual numbers of solutions with r.m.s.d. < 1 Å are specified in Table 3 ▸.

The success rate of solving the structures from such poor-quality data quickly dropped to about 2–50%; results of normal runs are shown in Fig. 8 ▸. Subsequently, these patterns were used step by step to solve the crystal structures with and without using the additional IMDs (Table 2 ▸) obtained from NMR crystallography.

The initial models for structure determination using the DS approach in *FOX* were taken from the structures solved in this work and were randomized in their mol­ecular positions and conformations. In this way, we obtained one randomized model for every compound that was used as a starting model for all testing runs to ensure the same starting conditions for all tests. The parallel tempering algorithm for the structure solution process was set to perform 1000 runs for every parameter set, each with 10^5^ trials. Parameters *s*_4_, σ and δ in 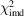 can be set individually, and their values will affect the final success rate of the calculation. The scale factor *s*_4_ gives the overall influence of 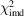 for the resulting CF, and σ in this parabolic formula is actually another representation of the scale factor. Therefore, for simplicity in testing, only different values of the parameter *s*_4_ were tested, while the values of σ were set to 1 Å and the values of δ were set according to the precision of the ssNMR distances in Table 2 ▸. Four parameter sets were defined for every compound: one normal run that did not use the IMDs, and three that used the IMDs listed in Table 2 ▸ and differed only in the scale factor *s*_4_, which was set to 10^4^, 10^5^ and 10^6^. The aim was to estimate the influence of the scale factor on the structure solution process. Every result list was classified on the basis of the similarity to the reference structure obtained by single-crystal X-ray diffraction (SCXRD). The similarity was evaluated as the r.m.s.d. value of the minimal distances of atomic positions in the overlapped molecular clusters that also contain anions using modified code of *CrystalCMP* (Rohlíček & Skořepová, 2020 ▸).

The results show that using IMDs in the structure determination process resulted in comparable or higher success rates than without their use. There is a notable difference in the success rates between the data sets with FWHM = 0.5° and FWHM = 1.5°, where the maximal success rate was 1.2 to 2.4 and 2 to 2.8 times higher, respectively, compared with a normal run (Table 3 ▸). IMDs were more advantageous for low-resolution data sets where structure determination is rather difficult due to the lack of structural information in the PD data. In these situations, the additional structural restrictions helped overcome this problem and significantly increased the probability of finding the correct solutions. For the scale factor *s*_4_, we can conclude that a value that is too low may have almost no effect on the success rate, while a value that is too high may yield a worse result than some lower values of the scale parameter (Fig. 8 ▸). Although these findings are as expected, testing them on a larger data set could provide better insight into the effect of the scale parameter on the success rates. In the case of **3·BF4**, the distance F⋯C1 was estimated from the ssNMR experiment as 4.5 ± 0.5 Å, but the distance from SCXRD was found to be 5.112 (3) Å. The difference, including accuracy, is approximately 0.1 Å, resulting in slightly higher absolute C⋯F values for all individual correct solutions. However, its influence on the success rates compared with those for the **1·BF4** and **2·BF4** compounds is not notable (Table 3 ▸). The results are depicted in Fig. 8 ▸, where all results of every determination process were sorted on the basis of their similarity to the reference structure.

## 3. Conclusions

In this study, we have synthesized six iso­thio­uronium salts in the forms of bromides and tetra­fluoro­borates (**1·Br**, **2·Br**, **3·Br**, **1·BF4**, **2·BF4** and **3·BF4**). We have described them by IR and liquid NMR spectroscopies and, with the exception of the already published **3·Br** (Eigner, 2020 ▸), we have also described their crystal structures using SCXRD. Additionally, the three tetra­fluoro­borates (**1·BF4**, **2·BF4** and **3·BF4**) were analysed using a combination of ssNMR techniques, including various 1D and 2D correlation experiments.

After careful calibration of NMR data against known standards, a comprehensive set of interatomic ^19^F⋯^13^C, ^11^B⋯^11^B, ^1^H⋯^1^H and ^13^C⋯^1^H distances were provided, together with a rough estimation of their precisions. The intermolecular distances between non-hydrogen atomic types were then used in the crystal structure determination process from the simulated PD data. The results confirm that the combination of ssNMR spectroscopy and PD analysis can be beneficial, and using intermolecular interactions as additional restrictions in crystal structure determination increases the probability of finding the correct solution.

This study underscores the synergistic advantages of combining experimental and computational approaches, thereby extending the utility of NMR crystallography and PD in elucidating the structures of challenging compounds, where every piece of additional structural information can be crucial for obtaining the structural model. The choice of structurally simple compounds allowed us to avoid the difficulties that the analysis of complex compounds entails. For more complex compounds such as solvates, cocrystals or complex compounds with many symmetry-independent molecules, both ssNMR and X-ray PD analysis will be correspondingly more complicated than for simple substances. However, we believe that the study of such compounds will be the next step offered by this approach.

## 4. Related literature

For further literature related to the supporting information, see Betteridge *et al.* (2003 ▸), Brandenburg (1999 ▸), Brown *et al.* (2004 ▸), Brown & Spiess (2001 ▸), Brus (2000 ▸), Edén *et al.* (2006 ▸), Hohwy *et al.* (1999 ▸), Langer *et al.* (1999 ▸), Palatinus & Chapuis (2007 ▸), Petříček *et al.* (2023 ▸), Rigaku (2020 ▸), Rohlíček & Hušák (2007 ▸), Salager *et al.* (2009 ▸), Schnell *et al.* (2001 ▸) and Wang *et al.* (2009 ▸).

## Supplementary Material

Crystal structure: contains datablock(s) global, 1Br_3489_VE018, 1BF4_VE_2A, 2Br_VE038, 2BF4_VE_8A, 3BF4_11A. DOI: 10.1107/S1600576724012378/ui5023sup1.cif

Structure factors: contains datablock(s) I. DOI: 10.1107/S1600576724012378/ui50231Br_3489_VE018sup2.hkl

Structure factors: contains datablock(s) I. DOI: 10.1107/S1600576724012378/ui50231BF4_VE_2Asup3.hkl

Structure factors: contains datablock(s) I. DOI: 10.1107/S1600576724012378/ui50232Br_VE038sup4.hkl

Structure factors: contains datablock(s) I. DOI: 10.1107/S1600576724012378/ui50232BF4_VE_8Asup5.hkl

Structure factors: contains datablock(s) I. DOI: 10.1107/S1600576724012378/ui50233BF4_11Asup6.hkl

Additional experimental details, plus extra figures and tables. DOI: 10.1107/S1600576724012378/ui5023sup7.pdf

Crystallographic data: https://doi.org/10.57680/asep.0605079

CCDC references: 2383958, 2383959, 2383960, 2383961, 2383962

## Figures and Tables

**Table 1:** Summary of the single-crystal X-ray diffraction data, with information on data collection, reduction and refinement

	**1·Br**	**1·BF4**	**2·Br**	**2·BF4**	**3·BF4**
Formula	C_10_H_13_N_2_S·Br	C_10_H_13_N_2_S·BF4	C_9_H_13_N_2_S·Br	C_9_H_13_N_2_S·BF_4_	C_12_H_13_N_2_S·BF_4_
*M*_r_	273.2	280.1	261.2	268.1	304.1
Crystal system	Monoclinic	Monoclinic	Monoclinic	Monoclinic	Monoclinic
Space group	*P*2_1_/*n*	*P*2_1_/*c*	*P*2_1_/*c*	*P*2_1_	*P*2_1_
*T* (K)	120	95	95	120	95
*a* (Å)	7.9876 (2)	5.6817 (4)	14.4689 (6)	5.6075 (2)	5.8011 (3)
*b* (Å)	8.2176 (2)	7.4235 (5)	6.1901(3)	7.7882 (3)	7.4127 (6)
*c* (Å)	17.1250 (4)	29.057 (2)	13.3423 (7)	13.8649 (6)	15.2291 (9)
β (°)	91.141 (2)	91.387 (6)	115.672 (4)	95.836 (3)	90.776 (5)
*V* (Å^3^)	1123.84 (5)	1225.20 (14)	1077.03 (10)	602.37 (4)	654.82 (7)
*Z*	4	4	4	2	2
*D*_calc_	1.615	1.518	1.611	1.478	1.542
μ (mm^−1^)	6.45	2.70	6.67	2.71	2.57
Crystal size (mm)	0.55 × 0.11 × 0.06	0.80 × 0.60 × 0.06	0.21 × 0.14 × 0.06	0.58 × 0.39 × 0.22	0.43 × 0.25 × 0.03
θ_min_, θ_max_ (°)	5.17, 67.37	3.04, 74.71	3.39, 74.15	3.20, 67.52	2.90, 74.75
θ_full_ (98%) (°)	67.37	67.68	72.34	67.52	74.75
Measured reflections	13275	3923	3712	5004	8858
Independent reflections	2016	2368	2111	2130	2640
*R*_int_	0.031	0.035	0.017	0.016	0.035
Observed reflections *I* > 3σ(*I*)	1903	2099	1998	2101	2595
*R*[*F*^2^ > 3σ(*F*^2^)]	0.0241	0.0788†	0.0206	0.0291	0.0337
*wR*[*F*^2^ > 3σ(*F*^2^)]	0.0723	0.2211†	0.0583	0.0776	0.0960
*R*(all)	0.0258	0.0834	0.0220	0.0293	0.0341
*wR*(all)	0.0737	0.2236	0.0601	0.0779	0.0964
*S*	1.43	1.04	1.11	1.64	1.86
Parameters	133	171	130	175	194
Restraints	2	8	4	4	4
Δρ_min_, Δρ_max_ (e^−^ Å^−3^)	−0.34, 0.60	−0.71, 0.82	−0.22, 0.27	−0.17, 0.31	−0.38, 0.17
CCDC number	2383959	2383958	2383961	2383960	2383962

† For these values, [*F*^2^ > 2σ(*F*^2^)].

**Table 2:** Intermolecular distances (in ångströms) obtained from ssNMR measurements and their comparison with distances measured from the single-crystal X-ray diffraction (SCXRD) model that satisfy the ssNMR range In the case of the F⋯C distances in the disordered crystal structure **2·BF4**, the upper rows are calculated distances between C and F of the major disorder and the bottom rows are for the minor disorder. In the case of the B⋯B distances, this is not recognized in the table and all distances are given together.

	ssNMR	SCXRD
**1·BF4**		
F⋯C2,6	4.9 ± 0.4	3.416 (6)†, 4.134 (7)†, 4.785 (7), 4.837 (6), 5.076 (6)
F⋯C7	3.4 ± 0.4	3.287 (8), 3.257 (6)
F⋯C8	3.8 ± 0.4	3.594 (7), 3.692 (8), 3.693 (7), 3.903 (6)
F⋯C9,10	3.1 ± 0.4	3.092 (6), 3.104 (7), 3.149 (7), 3.237 (7), 3.246 (6), 3.276 (6), 3.282 (6)
B⋯B	4.8 ± 0.4	5.073 (8)

**2·BF4**		
F⋯C1	4.2 ± 0.4	4.15 (3), 4.256 (16), 4.26 (2), 4.575 (17)
		4.282 (18), 4.523 (19), 4.24 (3)
F⋯C4	4.6 ± 0.4	4.523 (16), 4.79 (2), 4.99 (2)
		4.62 (2), 4.83 (3)
F⋯C7	3.2 ± 0.4	3.392 (19), 3.45 (2)
		3.31 (2), 3.43 (3)
F⋯C8	3.3 ± 0.4	3.63 (2), 3.63 (3)
		3.43 (2), 3.60 (3)
B⋯B	5.0 ± 0.3	5.11 (4), 4.97 (4), 5.00 (4), 4.87 (4)
	5.5 ± 0.3	5.41 (2), 5.61 (2), 5.607 (16)

**3·BF4**		
F⋯C1	4.5 ± 0.5	5.112 (3)†
F⋯C3	3.4 ± 0.4	3.388 (2)
F⋯C11	3.2 ± 0.4	3.186 (3)
F⋯C12	3.4 ± 0.4	3.406 (2), 3.409 (3), 3.493 (3), 3.579 (3)
B⋯B	4.5 ± 0.4	4.698 (4)
	5.8 ± 0.4	5.801 (3)

† This is the closest *A*⋯*B* distance but does not correspond to the distance measured by ssNMR.

**Table 3:** Number of solutions with r.m.s.d. difference < 1 Å from the reference structure, and their success rate multiplicity in parentheses, compared with the number of solutions of normal runs

	**1·BF4**		**2·BF4**		**3·BF4**	
	FWHM 0.5°	FWHM 1.5°	FWHM 0.5°	FWHM 1.5°	FWHM 0.5°	FWHM 1.5°
Normal run	221	23	168	77	526	126
*s*_4_ = 10^4^	258 (1.2×)	21 (0.9×)	259 (1.5×)	136 (1.8×)	602 (1.1×)	217 (1.7×)
*s*_4_ = 10^5^	282 (1.3×)	51 (2.2×)	410 (2.4×)	181 (2.4×)	651 (1.2×)	259 (2.1×)
*s*_4_ = 10^6^	212 (1.0×)	34 (1.5×)	399 (2.4×)	213 (2.8×)	584 (1.1×)	249 (2.0×)

## Data Availability

Crystallographic data can be obtained from the CCDC (https://www.ccdc.cam.ac.uk/structures/) using the CCDC numbers noted in Table 1 ▸ or from https://doi.org/10.57680/asep.0605079. NMR, IR and X-ray PD data are mostly presented in the supporting information. Alternatively, they can be requested from the correspondance author. The source code, including this approach and also the compiled versions of the program, can be found on the official web pages of the program *FOX*, https://github.com/vincefn/objcryst.
