# Supplementary material for: An advanced approach combining solid-state NMR with powder diffraction applied to newly synthesized iso­thio­uronium salts
Source: J Appl Crystallogr. 2025 Feb 11;58(Pt 2):321–32. doi: 10.1107/S1600576724012378 (PMC11957416; doi:10.1107/S1600576724012378)
Supplement: Supplementary file 7 [file j-58-00321-sup7.pdf]

JOURNAL OF APPLIED CRYSTALLOGRAPHY

Volume 58 (2025)

Supporting information for article:

An advanced approach combining solid-state NMR with powder diffraction applied to newly synthesized isothiouronium salts

Jan Rohlíček, Václav Eigner, Jiří Czernek and Jiří Brus

S1. IR and liquid NMR results

The FTIR spectra were measured using Nicolet 6700 spectrometer (Thermo-Nicolet, USA) using a GladiATR (PIKE, USA) ATR diamond and DTGS KBr detector.

The ^{1}H and ^{13}C NMR spectra in solutions were measured using a Bruker 600 AvanceIII Triple-resonance digital NMR spectrometer with actively shielded superconducting magnet UltraShield™ 14.1.

2-(Benzylthio)-4,5-dihydro-1H-imidazol-3-ium bromide (**1·Br**), white powder, yield: 4.477 g (93%).

^{1}H NMR (DMSO-d6, 600 MHz, 298 K) δ (ppm): 10.22 (b, 2H, NH), 7.47-7.30 (m, 5H, Ar-H), 4.54 (s, 2H, -S-CH2-), 3.87 (s, 4H, -NH-CH2-).

^{13}C NMR (DMSO-d6, 125 MHz, 298 K) δ (ppm):168.36, 134.82, 128.95, 128.85, 128.15, 45.08, 34.51.

IR (ATR, cm-1): 3063 (ν CN-H), 3028 (ν CN-H), 2959 (ν C-H2), 1551 (δ CN-H), 1530 (δ CN-H), 1495 (ν N-C-N), 1074 (ν N-C-N).

2-(Benzylthio)-4,5-dihydro-1H-imidazol-3-ium tetrafluoroborate (**1·BF4**) white powder, yield: 0.981 g (96%).

^{1}H NMR (DMSO-d6, 600 MHz, 298 K) δ (ppm): 10.17 (b, 2H, NH), 7.45-7.31 (m, 5H, Ar-H), 4.50 (s, 2H, -S-CH2-), 3.87 (s, 4H, -NH-CH2-).

^{13}C NMR (DMSO-d6, 125 MHz, 298 K) δ (ppm):168.38, 134.70, 128.91, 128.86, 128.17, 45.08, 34.44.

IR (ATR, cm-1): 3328 (ν CN-H), 2922 (ν C-H2), 1548 (δ CN-H), 1496 (ν N-C-N), 1056 (ν N-C-N), 1016 (ν BF4).

2-(4-Methylbenzyl)isothiouronium bromide (**2·Br**) white powder, yield: 3.824 g (90%).

^{1}H NMR (DMSO-d6, 600 MHz, 298 K) δ (ppm): 9.07 (b, 4H, NH2), 7.32-7.16 (dd, 4H, Ar-H), 4.46 (s, 2H, -S-CH2-), 2.28 (s, 3H, -CH3).

^{13}C NMR (DMSO-d6, 125 MHz, 298 K) δ (ppm): 169.01, 137.32, 131.75, 129.31, 128.87, 34.05, 20.66.

IR (ATR, cm-1): 3398 (ν N-H2), 3273 (ν N-H2), 2912 (ν C-H2), 1633 (δ N-H2), 1618 (δ N-H2), 1513 (ν N-C-N), 1072 (ν N-C-N).

2-(4-Methylbenzyl)isothiouronium tetrafluoroborate (**2·BF4**) white powder, yield: 0.893 g (87%).

^{1}H NMR (DMSO-d6, 600 MHz, 298 K) δ (ppm): 9.01 (b, 4H, NH2), 7.31-7.16 (dd, 4H, Ar-H), 4.42 (s, 2H, -S-CH2-), 2.29 (s, 3H, -CH3).

^{13}C NMR (DMSO-d6, 125 MHz, 298 K) δ (ppm): 168.93, 137.37, 131.67, 129.34, 128.84, 34.04, 20.65.

IR (ATR, cm-1): 3422 (ν N-H2), 3348 (ν N-H2), 3251 (ν N-H2), 3195 (ν N-H2), 2920 (ν C-H2), 1650 (δ N-H2), 1517 (ν N-C-N), 1021 (ν BF4).

2-(Naphthalen-2-ylmethyl)isothiouronium bromide (**3·Br**), white powder, yield: 0.704 g (87%).

^{1}H NMR (DMSO-d6, 600 MHz, 298 K) δ (ppm): 9.23 (b, 2H, NH2), 9.01 (b, 2H, NH2), 8.00-7.86 (m, 4H, Ar-H), 7.59-7.50 (m, 3H, Ar-H), 4.46 (s, 2H, -S-CH2-).

^{13}C NMR (DMSO-d6, 125 MHz, 298 K) δ (ppm): 168.85, 132.67, 132.49, 132.33, 128.54, 127.72, 127.63, 126.72, 126.62, 126.46, 34.55.

IR (ATR, cm-1): 3175 (ν N-H2), 3061 (ν N-H2), 2837 (ν C-H2), 1662 (δ N-H2), 1645 (δ N-H2), 1509 (ν N-C-N), 1075 (ν N-C-N).

2-(Naphthalen-2-ylmethyl)isothiouronium tetrafluoroborate (**3·BF4**), white powder, yield: 0.242 g (95%).

^{1}H NMR (DMSO-d6, 600 MHz, 298 K) δ (ppm): 9.06 (b, 4H, NH2), 7.97-7.86 (m, 4H, Ar-H), 7.57-7.50 (m, 3H, Ar-H), 4.64 (s, 2H, -S-CH2-).

^{13}CNMR (DMSO-d6, 125 MHz, 298 K) δ (ppm): 168.78, 132.67, 132.43, 132.33, 128.56, 127.69, 127.62, 126.69, 126.63, 126.47, 34.54.

IR (ATR, cm-1): 3421 (ν N-H2), 3346 (ν N-H2), 3253 (ν N-H2), 3193 (ν N-H2), 2839 (ν C-H2), 1650 (δ N-H2), 1510 (ν N-C-N), 1024 (ν BF4).

The differences among the IR spectra are quite pronounced, see Fig. S1. In the cases of the isothiouronium bromides the broad peaks of N-H stretching vibrations are observed and the bending N-H vibrations are split into two peaks. In the samples of tetrafluoroborates the N-H stretching vibrations peaks are significantly sharper, and in the cases of **2·BF4** and **3·BF4** four distinct peaks appeared, while the bending N-H vibration peaks are merged into one peak and broad peak at 1016, 1021 and 1024 in **1·BF4**, **2·BF4** and **3·BF4** respectively, related to stretching vibrations of the BF_4 anion.

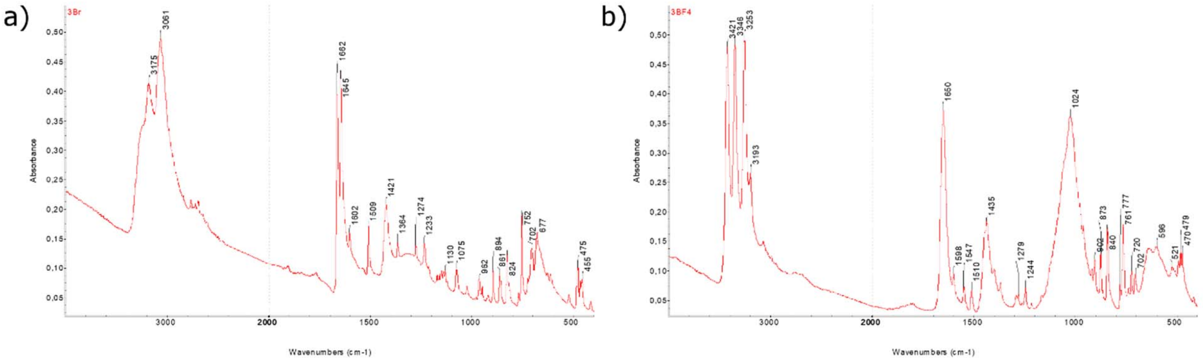

Figure S1 The IR spectra of a) **3·Br** and b) **3·BF4**.

S2. Experimental parameters for solid-state NMR spectroscopy

Solid-state NMR spectra were measured at 11.7 T using a Bruker Avance III HD 500 US/WB NMR spectrometer (Karlsruhe, Germany, 2013), and at 16.4 T using a Bruker Avance NEO 700 SB NMR spectrometer (Karlsruhe, Germany, 2021). In all cases, powdered samples were placed in the 3.2 mm ZrO2 rotors. The magic angle spinning frequency used for the spectra measurements was 10-20 kHz. The following techniques were applied: *i*) one-dimensional (1D) ^{1}H CRAMPS (DUMBO) [1] ^{19}F MAS, ^{11}B MAS, ^{13}C CP/MAS, ^{15}N CP/MAS and ^{19}F-^{13}C CP/MAS NMR experiments; *ii*) two-dimensional (2D) ^{1}H-^{1}H CRAMPS NMR experiment with DUMBO homodecoupling in both detection periods; *iii*) 2D ^{1}H-^{1}H double-quantum/single-quantum (DQ/SQ) CRAMPS NMR [2] experiment with SPC5 DQ recoupling [3] and DUMBO homodecoupling; 2D ^{1}H-^{13}C FSLG HETCOR experiments [4] and 2D ^{11}B-^{11}B double-quantum (DQ) experiments [5–8] with the $BR2^{1}_{2}$ recoupling sequence [9,10] (2D ^{11}B-^{11}B DQ/SQ $BR2^{1}_{2}$ MAS NMR). For the measurement of ^{19}F-^{13}C CP/MAS NMR spectra a special double-resonance probehead equipped with a duplexer allowing to perform triple resonance F/H/X experiments with simultaneous ^{1}H and ^{19}F decoupling during the acquisition of ^{13}C signals was used. Frictional heating [11,12] of the spinning samples was compensated by active cooling, and the temperature calibration was performed with $Pb(NO_3)_2$. The spectra were recorded at 295 K. For all experimental details, see Section S2 and Figures S2-S5.

Solid-state NMR spectra were measured at 11.7 T using a Bruker Avance III HD 500 US/WB NMR spectrometer (Karlsruhe, Germany, 2013), and at 16.4 T using a Bruker Avance NEO 700 SB NMR spectrometer (Karlsruhe, Germany, 2021). In all cases powdered samples were placed in the 3.2 mm ZrO_2 rotors. Magic angle spinning frequency used for the spectra measurements was 10-20 kHz.

The ^{13}C CP/MAS NMR spectra employing cross-polarization were acquired at 125.78 MHz using the standard pulse scheme at spinning frequency of 20 kHz. The recycle delay was 30 s, the cross-polarization contact time was 2 ms and the number of scans was 2000 - 4000. The strength of spin-locking fields $B_1(^{13}C)$ expressed in frequency units $\omega_1/2\pi=\gamma B_1$ was 64 kHz. The ^{13}C NMR chemical shift scale was referenced to α-glycine (176.03 ppm).

The ^{11}B MAS NMR spectra were acquired at 160.47 MHz; spinning frequency was $\omega_r/2\pi$= 20 kHz; 20° pulse width was 0.8 μs; recycle delay of 6 s; and the number of scans was 128. The ^{11}B NMR chemical shift scale was referenced to boric acid (16.43 ppm, signal with maximum intensity).

The ^{19}F MAS NMR spectra were acquired at 470.57 MHz; spinning frequency was $\omega_r/2\pi$= 20 kHz; 90° pulse width was 2.8 μs; recycle delay of 30 s; and the number of scans was 8. The ^{19}F NMR chemical shift scale was referenced to polytetrafluorethylene PTFE (-122 ppm).

The ^{15}N CP/MAS NMR spectra employing cross-polarization were acquired at 70.97 MHz using the standard pulse scheme at spinning frequency of 10 kHz. The recycle delay was 30 s, the cross-polarization contact time was 3 ms and the number of scans was 2000. The strength of spin-locking fields $B_1(^{15}N)$ expressed in frequency units $\omega_1/2\pi=\gamma B_1$ was 64 kHz. The ^{15}N NMR chemical shift scale was referenced to α-glycine (34.35 ppm).

The ^{1}H MAS NMR spectra with DUMBO homodecoupling were measured at 700.30 MHz at 10 kHz (MAS frequency) and number of scans 64-128. The initial 90° (^{1}H) pulse-length was 2.55 μs at power level 120 W, and DUMBO pulse length was 32 μs at power level 100 W. All parameters were optimized on glycine to reach maximum spectral resolution ($\Delta\nu(NH_3^+)$ = 380 Hz and $\Delta\nu(CH_2)$ = 290 Hz). The ^{1}H scale was calibrated with external standard – glycine (low-field NH_3 signal at 8.5 ppm and the high field α–H signal at 3.0 ppm.

The ^{19}F-^{13}C CP/MAS NMR spectra were recorded using a special double-resonance probe head equipped with a duplexer allowing to perform triple resonance F/H/X experiments with simultaneous ^{1}H and ^{19}F decoupling during the acquisition of ^{13}C signals. The recycle delay was 10 s, the cross-polarization contact time was ranging from 0.1 to 10 ms and the number of scans was 3072. The strength of spin-locking fields $B_1(^{13}C)$ and $B_1(^{19}F)$ expressed in frequency units $\omega_1/2\pi=\gamma B_1$ was 64 kHz. During the detection period of ^{13}C signals the dipolar decoupling SPINAL 64 and PIDEC 12 was applied to decouple ^{1}H and ^{19}F dipolar interactions, respectively.

^{1}H-^{1}H DQ/SQ DUMBO NMR correlation experiments: The 2D ^{1}H-^{1}H DQ/SQ DUMBO NMR correlation spectra were measured using the ^{1}H-^{1}H double-quantum (DQ) experiment employing the SPC5 recoupling sequence at spinning frequency $\omega_r/2\pi$ = 10 kHz (**Figure S2**). The recycle delay was 30 s, t_1 evolution period consisted of 128 increments each made of 32 scans. The DQ coherence excitation and reconversion consisted of 1-5 loops. The DUMBO decoupling was applied during both detection periods.

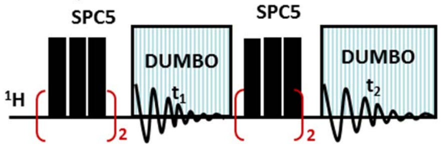

Figure S2 Schematic representation of 2D ^{1}H-^{1}H DQ/SQ DUMBO NMR experiment with SPC5 recoupling sequence.

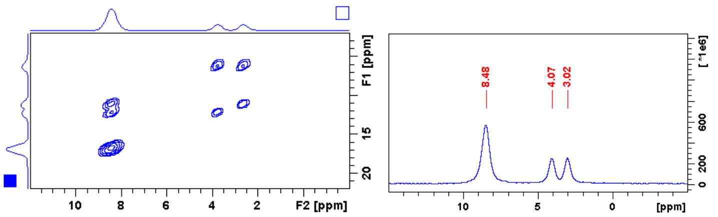

Figure S3 2D ^{1}H-^{1}H SQ/DQ DUMBO NMR and 1D ^{1}H DUMBO NMR spectra (10 kHz) of glycine.

2D ^{1}H-^{13}C FSLG HETCOR MAS NMR experiments: Two-dimensional (2D) ^{1}H-^{13}C HETCOR experiments were performed using the FSLG (Frequency Switched Lee-Goldburg) decoupling during the t_1 evolution period consisting of 64 increments each made of 128 scans with a dwell time of 42.6 μs (**Figure S4**). Rotation frequency was $\omega_r/2\pi$ = 11 kHz. The $B_1(^{1}H)$ field strength of FSLG and SPINAL-64 decoupling expressed in frequency units $\omega_1/2\pi=\gamma B_1$ was 89.3 kHz.

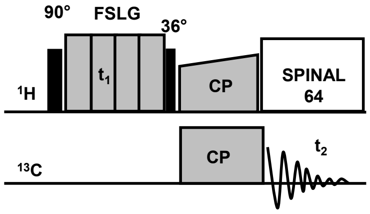

Figure S4 Schematic representation of 2D ^{1}H-^{13}C FSLG HETCOR MAS NMR experiment.

^{11}B-^{11}B DQ/SQ MAS NMR experiments: The ^{11}B-^{11}B DQ build-up dependences were measured using the ^{11}B-^{11}B double-quantum (DQ) experiment employing the $BR2^{1}_{2}$ recoupling sequence at spinning frequency $\omega_r/2\pi$ = 20 kHz (**Figure S5**). The DQ coherence excitation and reconversion consisted of 1-25 loops (duration of one loop was 200 μs). The recycle delay was 10 s. The dipolar decoupling SPINAL 64 was applied during both detection periods, and LG-cw decoupling was used during the build-up of DQ coherence. The first detection period t_1 was kept constant at 10 μs. Fast-amplitude modulation sequence (FAM) consisting of 2 loops was used for ^{11}B signal enhancement.

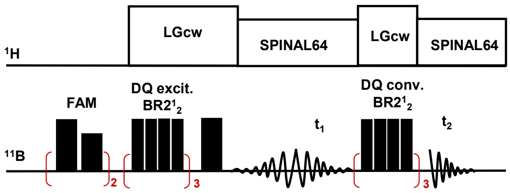

Figure S5 2D ^{11}B-^{11}B DQ-SQ experiment with FAM excitation and $BR2^{1}_{2}$ recoupling sequence.

S3. Single-crystal diffraction and geometry description of the crystal structures

The selected crystals were mounted on glass fibres and measured using a Rigaku OD Gemini (**1·Br**, **2·BF4**) with Atlas S2 CCD detector utilizing mirror collimated Cu *Kα* radiation (λ=1.54184 Å) from a sealed X-ray tube, or Rigaku OD Supernova (**1·BF4**, **2·Br**, **3·BF4**) with Atlas S2 CCD detector utilizing Cu *Kα* radiation (λ=1.54184 Å) from microfocused sealed X-ray tube. Samples **1·Br** and **2·BF4** were cooled by Oxford CryoSystems CryoJet5, while samples **1·BF4**, **2·Br** and **3·BF4** were cooled by Oxford CryoSystems CryoStream 800. The data integration, scaling and absorption correction were performed in CrysAlis PRO[13]. The phase problem was solved by charge flipping methods in Superflip[14]. The structure models **1·Br**, **2·Br**, **2·BF4** and **3·BF4** were refined by full-matrix least squares against F^2 in Jana2020[15], while structure model **1·BF4** was refined by full-matrix least squares against F^2 in Crystals[16]. The hydrogen atoms bonded to carbon were placed in calculated positions and refined with riding constraints, while hydrogen atoms bonded to nitrogen atoms were refined with restrained geometry. In both cases the U_{iso}(H)=1.2-1.5U_{eq}(C,N). In the case of **2·BF4**, tetrafluoroborate anion was found to be disordered over two positions. The disorder was modelled using molecular refinement, using a single model for both positions without restraints. The sum of occupancies was constrained to 1, resulting in final occupancy ratio 675(7):325(7). The MCE[17] was used for the visualization of electron density maps. The molecular graphics were displayed in Diamond 3[18] and Mercury 4.0[19].

Table S1 Selected bond lengths in studied compounds.

Structure	C(Ar)-C(Me) (Å)	C(Me)-S (Å)	S-C(iTh) (Å)	C(iTh)-N1 (Å)	C(iTh)-N2 (Å)
**1·Br**	1.501(3)	1.835(2)	1.734(2)	1.313(3)	1.325(3)
**1·BF4**	1.506(7)	1.831(5)	1.714(5)	1.314(7)	1.332(7)
**2·Br**	1.506(2)	1.819(2)	1.7430(19)	1.320(2)	1.314(3)
**2·BF4**	1.507(3)	1.824(3)	1.7447(17)	1.311(3)	1.310(3)
**3·Br**	1.495(5)	1.855(3)	1.771(3)	1.302(4)	1.316(4)
**3·BF4**	1.506(3)	1.832(2)	1.742(2)	1.311(3)	1.322(3)

Table S2 Selected bond angles in studied compounds.

Structure	C(Ar)-C(Me)-S (°)	C(Me)-S-C(iTh) (°)	S-C(iTh)-N1 (°)	S-C(iTh)-N2 (°)
**1·Br**	112.35(14)	103.44(10)	127.55(16)	120.62(16)
**1·BF4**	106.9(4)	101.1(2)	127.9(4)	121.6(4)
**2·Br**	114.74(13)	103.67(9)	122.91(16)	116.36(13)
**2·BF4**	107.06(15)	103.00(10)	122.14(17)	115.95(16)
**3·Br**	111.7(2)	96.99(16)	119.8(3)	118.4(3)
**3·BF4**	107.87(15)	102.18(10)	122.36(17)	116.34(16)

Table S3 Selected torsion angles in studied compounds.

Structure	C(Ar1)-C(Ar)-C(Me)-S (°)	C(Ar)-C(Me)-S-C(iTh) (°)	C(Me)-S-C(iTh)-N2 (°)
**1·Br**	91.2(2)	-89.48(15)	168.30(17)
**1·BF4**	99.8(5)	-172.8(4)	174.0(4)
**2·Br**	128.26(15)	-71.10(13)	165.25(15)
**2·BF4**	88.6(2)	169.73(13)	-169.61(15)
**3·Br**	91.8(4)	-68.2(2)	110.7(3)
**3·BF4**	-110.0(2)	163.25(15)	-166.02(17)

Since the studied compounds provide abundance of both hydrogen bond donors and acceptors in form of isothiouronium N–H groups and either bromide anions or fluorine of tetrafluoroborate, the hydrogen bonds play a significant role in structure packing, resulting in formation of hydrogen bonded chains and planes. As the bromide anion is spherical and can form hydrogen bonds in various directions the amount of hydrogen bonds formed varies among the structures. The lowest amount formed is observed in **1·Br** where the hydrogen bonding is constrained by the five-membered ring, resulting in formation of two N-H···Br hydrogen bonds and two weaker C-H···Br hydrogen bonds. The N-H···Br bonds and one C-H···Br bond connect molecules into chains, while the remaining C-H···Br bond connects the chains into ribbons in the direction of <0 1 0>, see Fig S6. In structures **2·Br** and **3·Br** the presence of four isothiouronium N-H groups results in higher impact of N-H···Br hydrogen bonds on the structure. In both cases formation of hydrogen bonded layers is observed. This is achieved by addition of $R_2^1(6)$ ring formed between two isothiouronium N-H groups and bromide anions, in addition to the chain observed in structure **1·Br**, see Fig S7. In structure **2·Br**, one of the chain forming hydrogen bonds is considerably weakened with the N···Br distance of 3.8 Å, this hydrogen bond is however supported by two weaker C-H···Br hydrogen bonds. Overall, the bromide anion is involved in three strong N-H···Br hydrogen bonds,

one weaker N-H···Br hydrogen bond and three C-H···Br hydrogen bonds. In structure **3·Br** all four N-H···Br hydrogen bonds are of comparable strengths, resulting in formation of just one weaker C-H···Br hydrogen bond and one chalcogen bond. Therefore, bromide anion participates in formation of six noncovalent interactions. For further information on non-covalent interactions see Table S4.

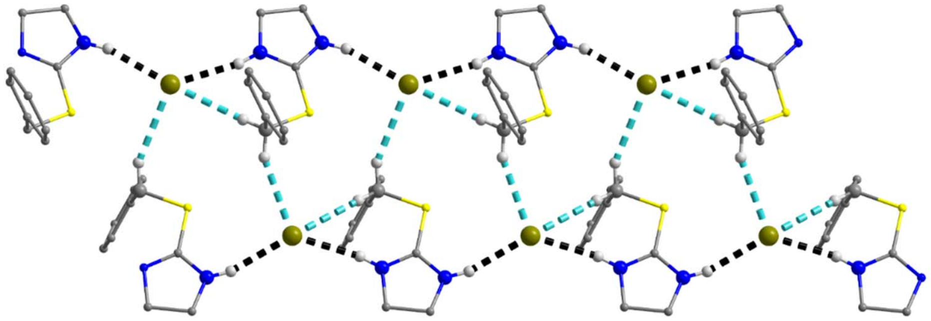

Figure S6 The ribbon formed in the structure **1·Br**, with N-H···Br hydrogen bonds depicted as black dashed lines and C-H···Br bonds depicted in light blue. The atoms drawn as spheres of arbitrary radii, hydrogen atoms not involved in depicted hydrogen bonding were omitted for clarity.

a) b)

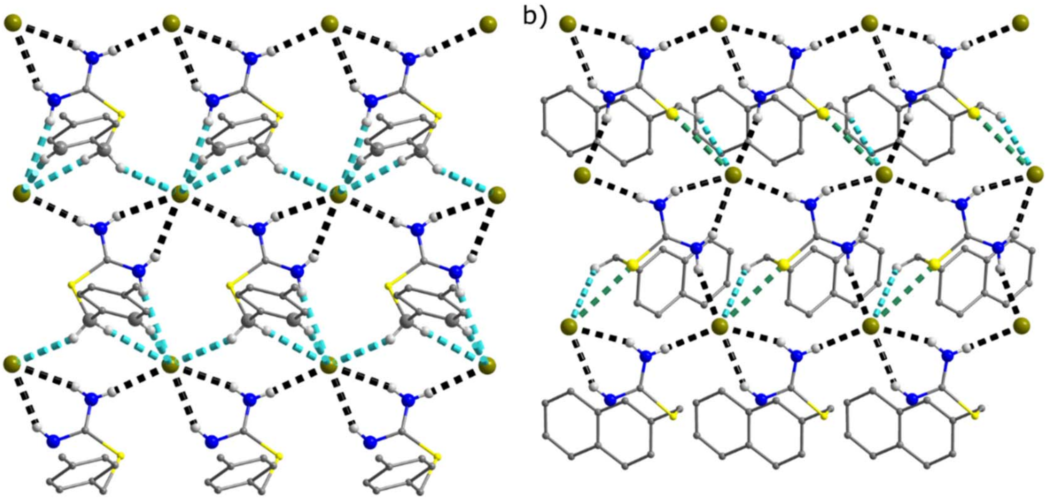

Figure S7 The layers formed in the structures a) **2·Br** and b) **3·Br**, with strong N-H···Br hydrogen bonds depicted as black dashed lines, weaker N-H···Br and C-H···Br bonds depicted in light blue and chalcogen bonds depicted in green. The atoms drawn as spheres of arbitrary radii, hydrogen atoms not involved in depicted hydrogen bonding were omitted for clarity.

Table S4 Selected non-covalent interactions in structures **1·Br**, **2·Br** and **3·Br**.

1·Br				
D–H···A	D–H (Å)	H···A (Å)	D···A (Å)	D–H···A (°)
N1–H1n1···Br1	0.860(17)	2.444(15)	3.2873(18)	167(2)
N2–H1n2···Br1*i*	0.860(11)	2.465(17)	3.3004(19)	164(3)
C7–H1c7···Br1	0.96	2.88	3.829(3)	169
C7–H2c7···Br1*ii*	0.96	2.93	3.826(2)	155
2·Br				
D–H···A	D–H (Å)	H···A (Å)	D···A (Å)	D–H···A (°)
N1–H1n1···Br1	0.86(2)	2.66(2)	3.4508(18)	153.1(17)
N1–H2n1···Br1*i*	0.86(2)	3.037(18)	3.8614(18)	161(2)
N2–H1n2···Br1	0.86(2)	2.630(16)	3.3938(15)	149(2)
N2–H2n2···Br1*ii*	0.860(12)	2.519(13)	3.3747(15)	173.6(18)
C2–H1c2···Br1*i*	0.96	3.05	3.9373(15)	155
C7–H1c7···Br1*i*	0.96	2.93	3.8766(14)	168
C7–H2c7···Br1*iii*	0.96	2.95	3.7296(14)	140
3·Br				
D–H···A	D–H (Å)	H···A (Å)	D···A (Å)	D–H···A (°)
N1–H1n1···Br1	0.86(3)	2.54(3)	3.331(3)	153(3)
N1–H2n1···Br1*i*	0.86(2)	2.49(2)	3.350(3)	180(3)
N2–H1n2···Br1*ii*	0.86(2)	2.55(3)	3.381(3)	163.8(13)
N2–H2n2···Br1	0.86(3)	2.68(3)	3.434(3)	147(3)
C11–H2c11···Br1*iii*	0.96	3.04	3.703(4)	128
D–S···A	D–S (Å)	S···A (Å)	D···A (Å)	D–S···A (°)
C12–S1···Br1*iii*	1.771(3)	3.5781(9)	5.341(3)	173.42(11)

Symmetry codes: **1·Br** (*i*) *x*, 1+*y*, *z*; (*ii*) 0.5-*x*, 0.5+*y*, 0.5-*z*; **2·Br** (*i*) *x*,0.5-*y*, -0.5+*z*; (*ii*) *x*, 1+*y*, *z*; (*iii*) *x*, 1.5-*y*, -0.5+*z*; **3·Br** (*i*) *x*, -1+*y*, *z*; (*ii*) *x*, 1.5-*y*, -0.5+*z*; (*iii*) *x*, 0.5-*y*, -0.5+*z*.

Among the tetrafluoroborate structures a similar structure packing is observed, most notably the presence of N-H···F hydrogen bonded chains in directions <1 1 0> and <1 $\bar{1}$ 0>, see Fig S8. In structure **1·BF4** the chains do not intersect but are connected by numerous weak C-H···F and C-H···π hydrogen bonds. This most likely is not caused by the five membered ring blocking the formation of forward-facing hydrogen bonds, as the hydrogen bonds are replaced by tetrel bonds and the interplanar angle tetrafluoroborate anion and isothiouronium group are very similar in all the structures, with values of 60.0(4), 53(2) and

58.7(2) for structures **1·BF4**, **2·BF4** and **3·BF4** respectively. Therefore, this has to be the result of weaker non-covalent interactions within the crystal structure. The tetrafluoroborate anions form two N-H···F hydrogen bonds, five weak C-H···F hydrogen bonds and three tetrel bonds. Out of these interactions, all but one weak C-H···F hydrogen bond form layers within the structure, these layers have hydrogen bond chains going in the same direction. The layers are connected by the afore-mentioned C-H···F hydrogen bond and N-H···F the C-H···π interactions between the phenyl rings. Since the C-H···F hydrogen bond is formed with hydrogen atom in para-position to the methylene bridge, it has only a small effect on the phenyl ring rotation; therefore, the C-H···π interactions are the root cause for formation of both diagonal hydrogen bonded chains in the structure. In structures **2·BF4** and **3·BF4**, both <1 1 0> and <1 $\bar{1}$ 0> hydrogen bonded chains intersect, resulting in formation of hydrogen bonded planes and lowering the significance of C-H···π interactions on structure packing. The C-H···π interactions still connect the created layers, however, it no longer affects the hydrogen bond directionality. With freeing two isothiouronium N-H groups the decreased number of weaker non-covalent interactions formed by tetrafluoroborate anion is observed, with only two C-H···F hydrogen bonds formed in structure **2·BF4** and three C-H···F hydrogen bonds and one chalcogen bond formed in structure **3·BF4**. For further information on non-covalent interactions see Table S5.

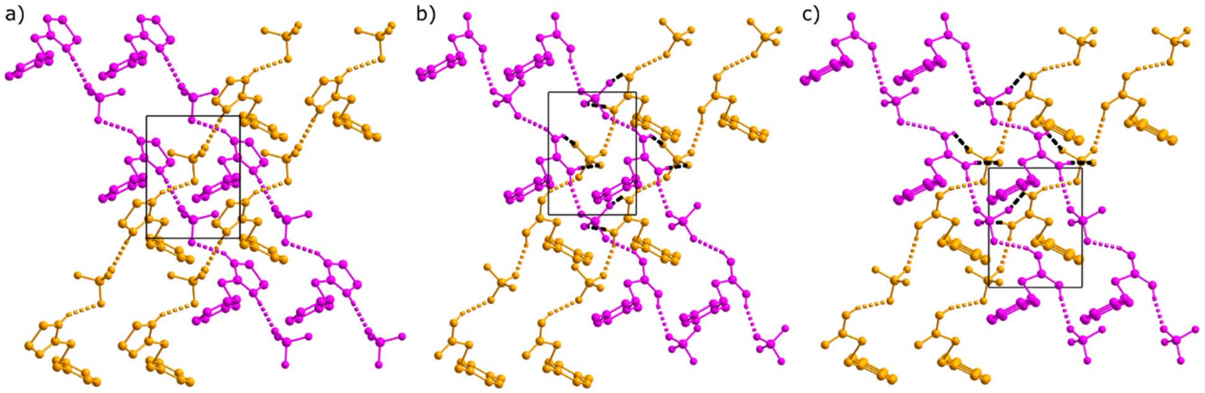

Figure S8 The hydrogen bonded chains in structures a) **1·BF4**, b) **2·BF4** and c) **3·BF4** viewed in direction of *c* axis. The chains in directions <1 1 0> and <1 $\bar{1}$ 0> depicted in magenta and orange respectively. The hydrogen bonds within the chain depicted in the same colour, the hydrogen bonds connecting the chains depicted in black.

Table S5 Selected non-covalent interactions in structures **1·BF4**, **2·BF4** and **3·BF4**.

1·BF4				
D–H···A	D–H (Å)	H···A (Å)	D···A (Å)	D–H···A (°)
N1–H11···F4[i]	0.86(4)	2.04(4)	2.870(6)	160(4)
N2–H21···F2[ii]	0.85(5)	2.22(5)	3.002(7)	152(5)
C4–H41···F2[iii]	0.93	2.66	3.534(7)	157
C6–H61···F4	0.93	2.51	3.416(6)	135
C7–H72···F3	0.97	2.43	3.288(8)	166
C9–H91···F1[iv]	0.97	2.57	3.517(7)	148
C9–H92···F1[v]	0.97	2.52	3.276(6)	164
D–S···A	D–S (Å)	S···A (Å)	D···A (Å)	D–S···A (°)
N2–C9···F3[vi]	1.466(7)	3.104(7)	4.498(7)	158.1(4)
H92–C9···F2[iv]	0.97	3.149(7)	4.05	155
N1–C10···F1[vi]	1.469(7)	3.092(6)	4.524(6)	164.3(4)
2·BF4				
D–H···A	D–H (Å)	H···A (Å)	D···A (Å)	D–H···A (°)
N1–H1n1···F3b[i]	0.86(2)	2.02(3)	2.88(3)	172(3)
N1–H2n1···F1b[ii]	0.860(16)	2.09(3)	2.86(3)	149(2)
N2–H1n2···F4b[iii]	0.86(2)	2.12(4)	2.94(3)	158(2)
N2–H2n2···F2b[i]	0.86(2)	2.23(3)	2.99(3)	148(3)
N2–H2n2···F3b[iv]	0.86(2)	2.51(5)	3.15(3)	132(2)
C2–H1c2···F1b[v]	0.96	2.42	3.372(16)	170
C7–H1c7···F2b[v]	0.96	2.65	3.43(3)	138
3·BF4				
D–H···A	D–H (Å)	H···A (Å)	D···A (Å)	D–H···A (°)
N1–H1n1···F1[i]	0.860(17)	2.048(16)	2.883(3)	163(3)
N1–H2n1···F2[ii]	0.86(2)	2.06(2)	2.901(2)	167(3)
N1–H2n1···F3[ii]	0.86(2)	2.54(2)	3.104(2)	124(2)
N2–H1n2···F2[iii]	0.86(2)	2.43(3)	3.094(4)	134(3)
N2–H1n2···F3[ii]	0.86(2)	2.35(3)	3.043(3)	138(3)
N2–H2n2···F4[iv]	0.86(2)	2.14(2)	2.964(3)	160(2)
C3–H1c3···F1[v]	0.96	2.43	3.388(2)	174
C6–H1c6···F4[vi]	0.96	2.63	3.463(3)	146
C11–H1c11···F1[i]	0.96	2.59	3.186(3)	121
D–S···A	D–S (Å)	S···A (Å)	D···A (Å)	D–S···A (°)
C11–S1···F4[iv]	1.832(2)	3.2349(17)	5.045(3)	168.95(8)

Symmetry codes: **1·BF4** (*i*) 1+*x*, *y*, *z*; (*ii*) *x*, -1+*y*, *z*; (*iii*) 1-*x*, -0.5+*y*, 1.5-*z*; (*iv*) 1+*x*, -1+*y*, *z*; (*v*) 1-*x*, -*y*, 1-*z*; (*vi*) 2-*x*, -*y*, 1-*z*; **2·BF4** (*i*) 1+*x*, *y*, *z*; (*ii*) 2-*x*, -0.5+*y*, 1-*z*; (*iii*); 1-*x*, 0.5+*y*, 1-*z*; (*iv*) 2-*x*, 0.5+*y*, 1-*z*; (*v*) 1-*x*, -0.5+*y*, 1-*z*; **3·BF4** (*i*) -*x*, -0.5+*y*, 1-*z*; (*ii*) *x*, *y*, 1+*z*; (*iii*) -*x*, 0.5+*y*, 1-*z*; (*iv*) 1-*x*, 0.5+*y*, 1-*z*; (*v*) 1-*x*, -0.5+*y*, 1-*z*; (*vi*) 1+*x*, *y*, *z*.

S4. Details of DFT calculations of isotropic chemical shifts

The crucial step in reliable internuclear distance estimation is to have as complete a signal assignment as possible. Therefore, we performed high-level quantum chemical geometry optimization of the previously refined single-crystal X-ray structures and calculated chemical shifts. As shown below, in all cases, the correlation between the experimentally determined ^{1}H and ^{13}C isotropic chemical shifts and the corresponding DFT-calculated chemical shielding parameters are very good in terms of the root-mean-square-deviation values, which lie between 0.18 to 0.29 ppm for ^{1}H and between 1.18 to 2.08 ppm for ^{13}C (Figures S10-S11). Therefore, the signal assignment was taken as obtained by these calculations, and internuclear distances were analysed accordingly.

During the geometrical optimizations and the predictions of the NMR chemical shielding values, the CASTEP settings were used which are consistent with ‘Fine’ level of accuracy of Materials Studio 2019 software. In particular, the cut-off value of 550 eV for the plane-waves energy was applied together with the default Monkhorst–Pack grids.

The values of the measured chemical shifts read out from the ^{1}H CRAMPS, ^{13}C CP/MAS and ^{1}H-^{13}C FSLG HETCOR spectra were assigned with the aid of the GIPAW-PBE calculations of the corresponding chemical shieldings. The best agreement was sought between the chemical shielding at known crystallographic positions and the corresponding chemical shift, whose value was chosen from among of a suitable subset of the measured data, while taking into account the experimentally established ^{1}H-^{13}C connectivities.

S4.1. Compound 3·BF4

Table S6 Experimental and DFT-calculated values of ^{13}C NMR chemical shifts of **3·BF4** compound.

#C	Exp ^{13}C, ppm	DFT-σ, ppm	DFT-δ, ppm
C11	35.78	133.2758	34.22
C3	125.72	44.9161	126.16
C5	127.16	42.8601	128.30
C6	127.16	43.5704	127.56
C7	127.16	43.2377	127.90
C2	127.8	42.4536	128.72
C8	127.8	42.1595	129.03
C4	129.21	40.6787	130.57
C10	129.21	40.8896	130.35
C1	132.01	40.7076	132.01
C9	132.01	40.0074	131.26
C12	172.77	3.5512	169.20

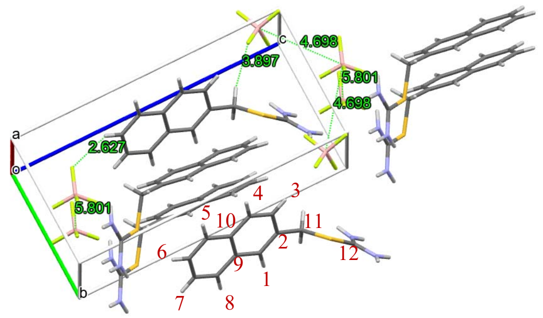

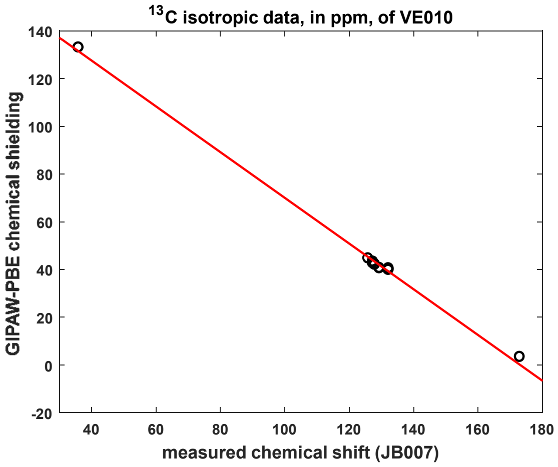

Figure S9 Correlation plot between the experimental and DFT-calculated ^{13}C NMR chemical shifts of **3·BF4**.

S4.2. Compound 2·BF4

Table S7 Experimental and DFT-calculated values of ^{13}C NMR chemical shifts of **2·BF4** compound.

^{13}C NMR chemical shifts and shielding			
#C	Exp ^{13}C, ppm	DFT-σ, ppm	DFT-δ, ppm
C1	127.14	44.6912	*127.3*
C2	129.70	40.6311	*131.4*
C3	130.42	39.1912	*132.8*
C4	139.11	29.6376	*142.4*
C5	131.08	39.1213	*132.9*
C6	131.08	38.6182	*133.4*
C7	34.94	134.3554	*37.6*
C8	172.32	3.6375	*168.4*
C9	21.98	150.3495	*21.7*

^{1}H NMR chemical shifts and shielding				
#Hcryst	#Hnmr	Exp ^{1}H, ppm	DFT-σ, ppm	DFT-δ, ppm
H1c2	H2	7.79	22.2487	*8.25*
H1c3	H3	5.62	24.7683	*5.73*
H1c5	H5	6.18	23.8743	*6.63*
H1c6	H6	4.97	25.5082	*4.99*
H1c7	H7	4.75	25.5228	*4.98*
H2c7	H7	3.50	26.5356	*3.96*
H1-3c9	H9	1.33	28.6988	*1.80*
H1n1	H10	*b 7.0-9.0*	22.5230	*7.98*
H2n1	H10	*b 7.0-9.0*	22.2609	*8.24*
H1n2	H11	*b 7.0-9.0*	20.8639	*9.64*
H2n2	H11	*b 7.0-9.0*	22.4951	*8.00*

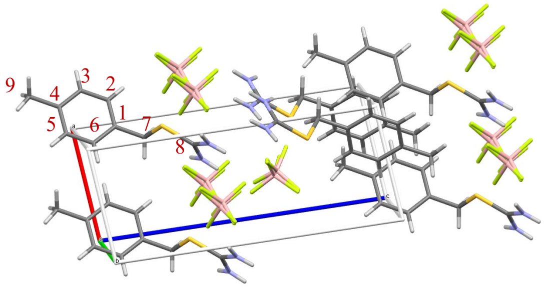

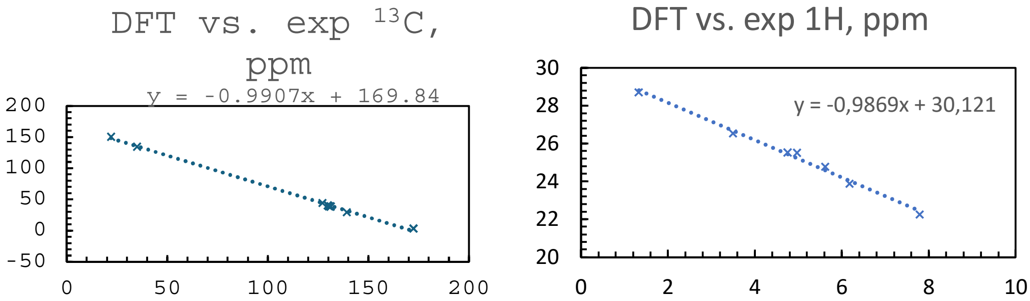

Figure S10 Correlation plot between the experimental ^{13}C NMR chemical shifts and DFT-calculated ^{13}C chemical shielding parameters, and the experimental ^{1}H NMR chemical shifts and DFT-calculated ^{1}H chemical shielding parameters obtained for **2·BF4** compound.

S4.3. Compound 1·BF4

Table S8 Experimental and DFT-calculated values of ^{13}C NMR chemical shifts of **1·BF4** compound.

^{13}C NMR chemical shifts and shielding			
#C	Exp ^{13}C, ppm	DFT-σ, ppm	DFT-δ, ppm
C1	130.17	40.2749	*132.5*
C2	131.12	38.8070	*134.0*
C3	130.04	40.0371	*132.8*
C4	129.45	41.3060	*131.5*
C5	129.17	41.1865	*131.6*
C6	129.17	41.1861	*131.6*
C7	35.47	133.9594	*38.8*
C8	170.86	6.2548	*166.5*
C9	46.75	125.5334	*47.3*
C10	46.75	124.3916	*48.4*

^{1}H NMR chemical shifts and shielding				
#Hcryst	#Hnmr	Exp ^{1}H, ppm	DFT-σ, ppm	DFT-δ, ppm
H22	H2	3.9	26.0417	*4.46*
H31	H3	6.73	23.2668	*7.23*
H41	H4	7.09	22.6986	*7.80*
H51	H5	3.9	25.8360	*4.66*
H61	H6	6.73	23.1238	*7.38*
H71	$H7^{1}$	2.85	27.2021	*3.30*
H72	$H7^{2}$	4.52	25.0607	*5.44*
H91	$H9^{1}$	4.34	25.0848	*5.42*
H92	$H9^{2}$	4.34	25.1755	*5.32*
H102	$H10^{1}$	4.34	25.5671	*4.93*
H101	$H10^{2}$	4.34	25.7444	*4.76*
H11	H11	8.21	21.4099	*9.09*
H21	N12	7.94	22.3999	*8.10*

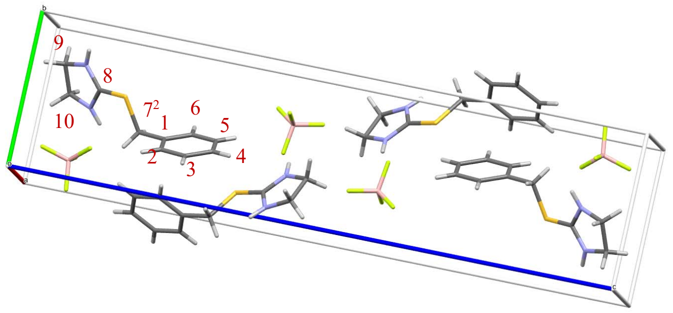

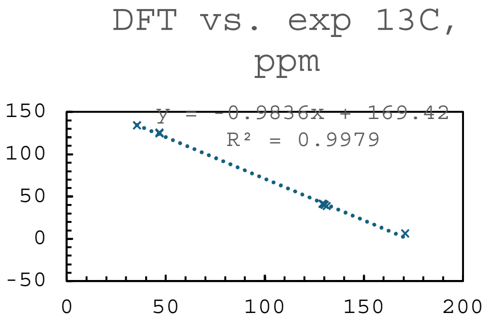

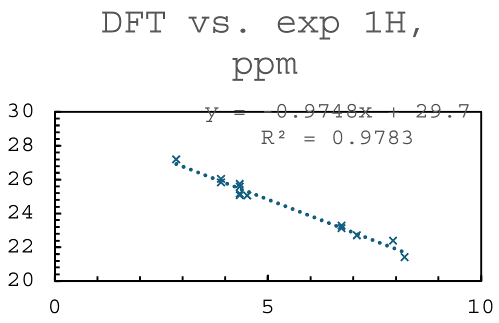

Figure S11. Correlation plot between the experimental ^{13}C NMR chemical shifts and DFT-calculated ^{13}C chemical shielding parameters, and the experimental ^{1}H NMR chemical shifts and DFT-calculated ^{1}H chemical shielding parameters obtained for **1·BF4** compound.

S5. Additional ss-NMR data

^{11}B MAS NMR spectra

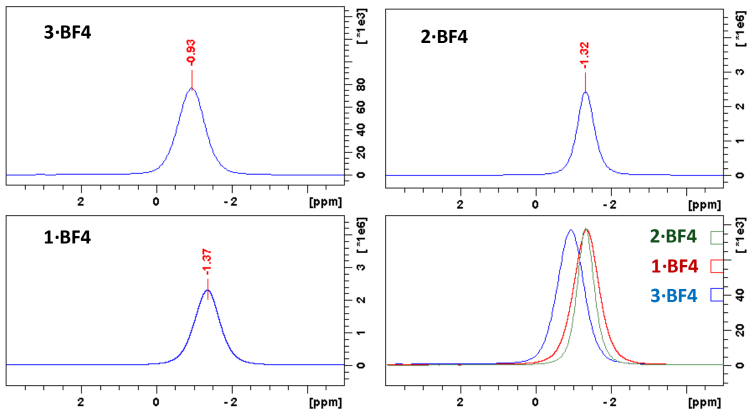

Figure S12 ^{11}B MAS NMR spectra of **3·BF4**; **2·BF4**; and **1·BF4** systems.

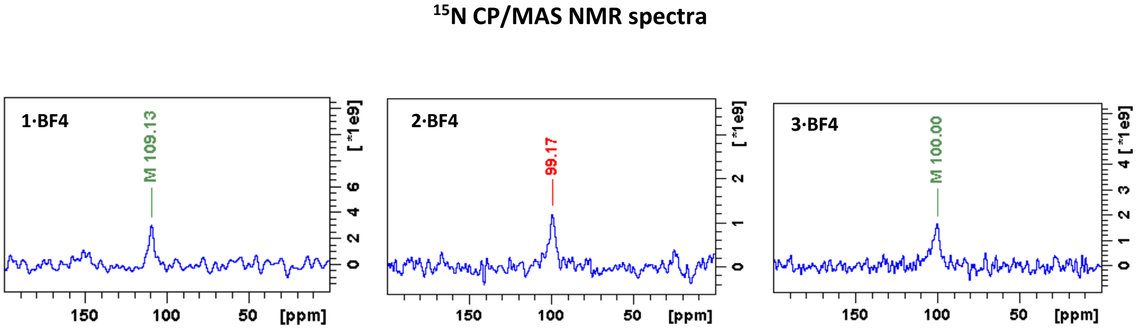

Figure S13 ^{15}N CP/MAS NMR spectra of **3·BF4**; **2·BF4**; and **1·BF4** systems.

S5.1. $^{13}C\{^{19}F\}$ CP/MAS NMR spectra and ^{19}F-^{13}C CP buildups for TFA

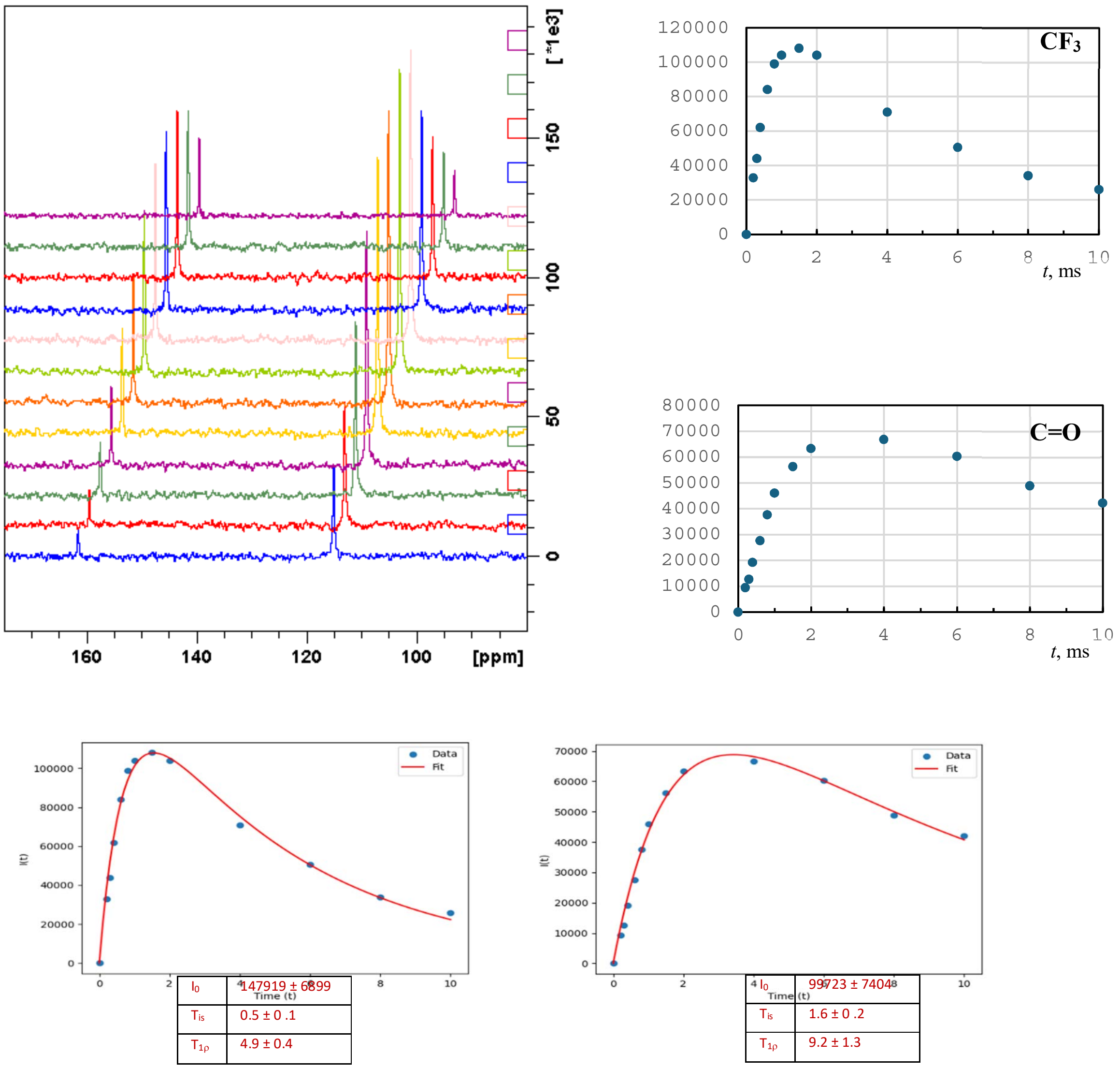

I_0	147919 ± 6899
T_{is}	0.5 ± 0 .1
$T_{1\rho}$	4.9 ± 0.4

I_0	99723 ± 74048
T_{is}	1.6 ± 0 .2
$T_{1\rho}$	9.2 ± 1.3

Figure S14 $^{13}C\{^{19}F\}$ CP/MAS NMR spectra of crystalline sodium trifluoroacetate (TFA) measured at different cross-polarization mixing times 0.2-10 ms; and the corresponding ^{19}F-^{13}C cross-polarization build-up curves created for CF3 and C=O carbon atoms.

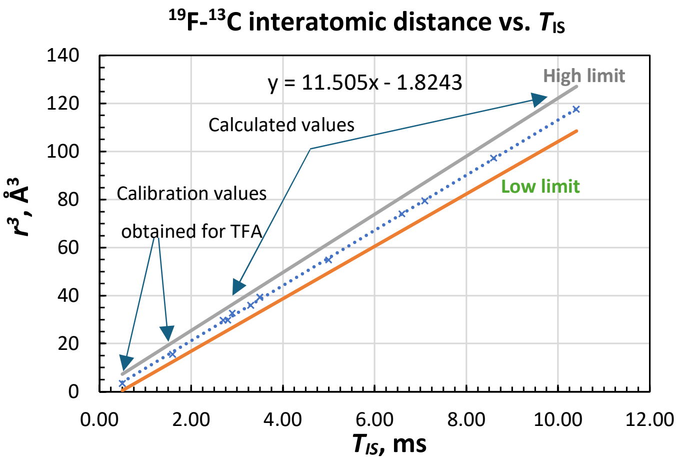

Figure S15 The calibration dependence between the time constant T_{IS} and the third power of ^{19}F-^{13}C interatomic distance r^3. The calibration values determined for known interatomic distances (crystalline sodium trifluoroacetate, TFA) are marked by arrows. Other values were calculated using the calibration function from the determined T_{IS} constants. Grey and orange lines represent upper and lower limits of interatomic distances calculated form the experimental error of the estimated T_{IS} constants (±0.5-0.7 ms).

S5.2. $^{13}C\{^{19}F\}$ CP/MAS NMR spectra and ^{19}F-^{13}C CP buildups for 3·BF4, 2·BF4 and 1·BF4

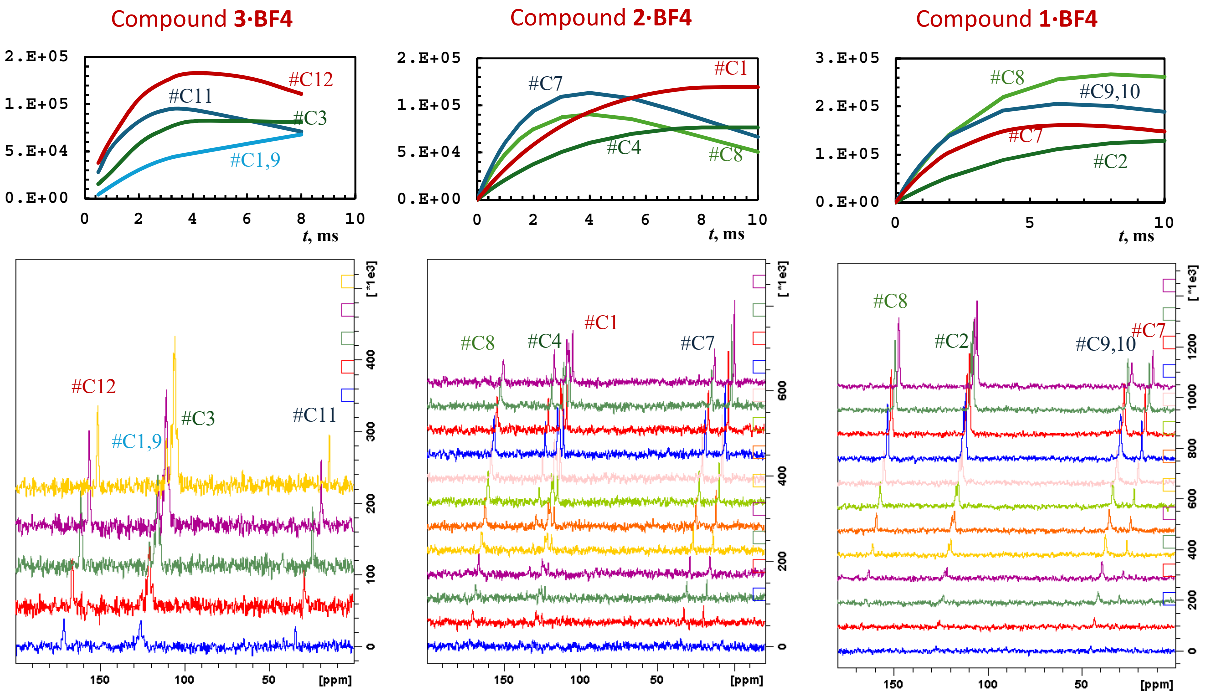

Figure S16 $^{13}C\{^{19}F\}$ CP/MAS NMR spectra of the systems **3·BF4**, **3·BF4** and **1·BF4** measured at different cross-polarization mixing times 0.2-10 ms (bottom panels); and the selected representative ^{19}F-^{13}C cross-polarization build-up curves (upper graphs).

Table S9 The set of $^{19}F...^{13}C$ cross-polarization time constants T_{IS} and the corresponding $^{19}F...^{13}C$ interatomic distances calculated using the calibration function (Fig S15). As a reference compound we used crystalline sodium trifluoroacetate in which the one-bond $^{19}F...^{13}C$ pair in CF_3 group with the distance 1.4 Å is characterized by the T_{IS} constants of ca. 0.5±0.1 ms and the two-bond $^{19}F...^{13}C$ pair (CF_3...CO) with the distance of ca. 2.5 Å is characterized by the T_{IS} constants of ca. 1.6±0.2 ms.

Spin pair		T_{IS}, ms	$^{19}F...^{13}C$ distances *r*, Å
**1·BF4**			
F…C8	-N-C-S-	5.0±0.5	3.8 ± 0.4 Å
F…C7	-CH2-S-	3.5±0.5	3.4 ± 0.4 Å
F…C9,10	-CH2-N-	2.7±0.5	3.1 ± 0.4 Å
F…C2,6	-CH=	10.4±0.7	4.9 ± 0.4 Å
**2·BF4**			
F…C8	-N-C-S-	3.3±0.5	3.3 ± 0.4 Å
F…C7	-CH2-S-	2.9±0.5	3.2 ± 0.4 Å
F…C4	-C=	8.6±0.7	4.6 ± 0.4 Å
F…C1	-C=	6.6±0.6	4.2 ± 0.4 Å
**3·BF4**			
F…C11	-C=	2.9±0.5	3.2 ± 0.4 Å
F…C12	-CH=	3.5±0.5	3.4 ± 0.4 Å
F…C3	-N-C-S-	3.5±0.5	3.4 ± 0.4 Å
F…C1	-CH=	7.8±0.7	4.5 ± 0.5 Å

Table S10 The set of $^{11}B\ldots^{11}B$ interatomic distances calculated according to the calibration function $r = 0.23t_m^{0.38}$, relating the recoupling time at maximum DQ coherence intensity and the interatomic $^{11}B\ldots^{11}B$ distance, *r*. As the recoupling time of maximum DQ coherence, t_m, can be determined only roughly with an experimental error of ca. ± 0.4 ms, which is given by the increment of recoupling loops used, the $^{11}B\ldots^{11}B$ interatomic distances are estimated with an experimental error of about ± 0.2 Å at least. However, also in this case the determined B…B distances must be considered as rough estimates. This is particularly because the evolution of double-quantum coherences of ^{11}B is affected by undecoupled interactions with ^{19}F spins. Therefore, we rather suppose that the interatomic distances are estimated with an experimental error of about ± 0.3-0.4 Å.

Spin pair	t_m, ms	$^{11}B\ldots^{11}B$ distances *r*, Å
**1·BF4**		
B…B	2.3 ± 0.5	4.8 ± 0.4 Å *)
**2·BF4**		
B…B	3.2 ± 0.4	5.3 ± 0.4 Å
		Bimodal distribution
		5.0 ± 0.3 Å
		5.5 ± 0.3 Å
**3·BF4**		
B…B	3.8 ± 0.4	5.8 ± 0.4 Å
	1.8 ± 0.4	4.5 ± 0.4 Å *)

*) In spite of our maximum effort we were not able to obtain high-quality ^{11}B-^{11}B DQ build-up dependence for **1·BF4** compound. Probably from unknown reasons the recorded dependence was contaminated by the residual single-quantum coherences or coherences resulted from the interactions with undecoupled ^{19}F spins.

S5.3. ^{1}H-^{1}H SQ/DQ CRAMPS NMR spectra for 3·BF4, 2·BF4 and 1·BF4

Compound **3·BF4**

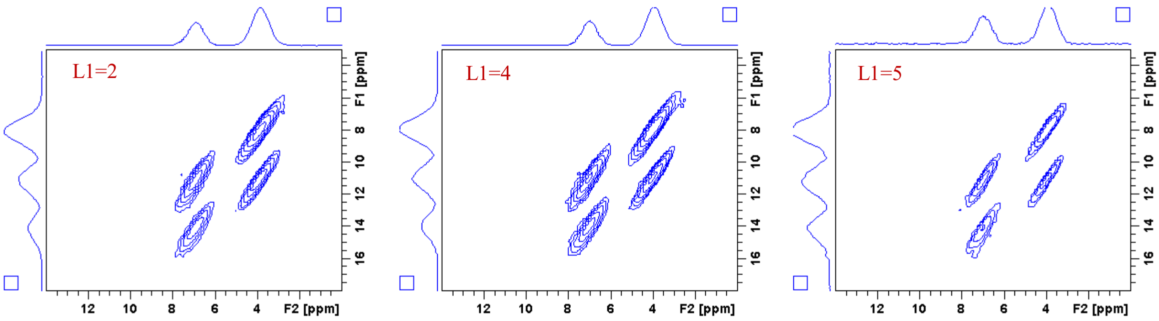

Figure S17 ^{1}H-^{1}H SQ/DQ CRAMPS NMR spectra of **3·BF4** measured at different recoupling loops 2; 4 and 5.

Compound **2·BF4**

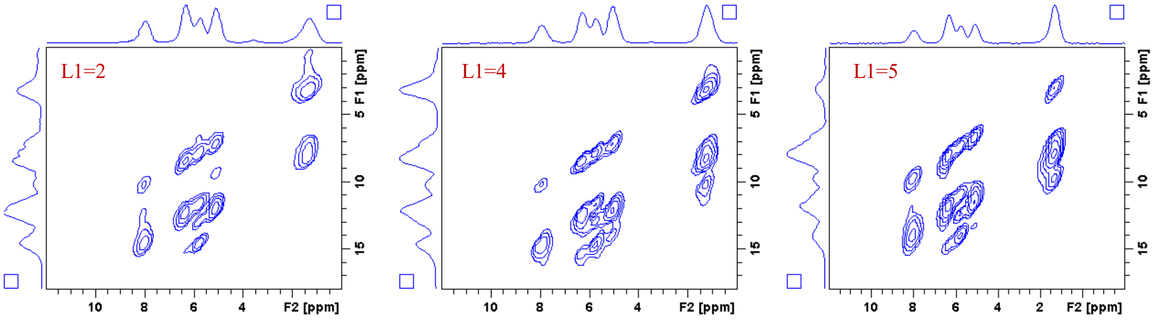

Figure S18 ^{1}H-^{1}H SQ/DQ CRAMPS NMR spectra of **2·BF4** measured at different recoupling loops 2; 4 and 5.

Compound **1·BF4**

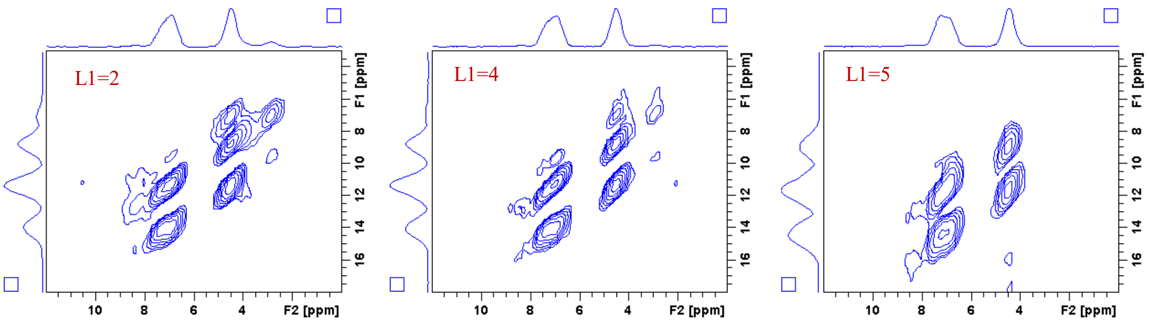

Figure S19 ^{1}H-^{1}H SQ/DQ CRAMPS NMR spectra of **1·BF4** measured at different recoupling loops 2; 4 and 5.

Table S11 The set of $^{1}H\ldots{}^{1}H$ interatomic distances, *r*, estimated from the intensity of autocorrelations signals detected at given recoupling time t_r (four recoupling loops)

Spin pair	t_r, μs	$^{1}H\ldots{}^{1}H$ distances *r*, Å
**2·BF4**		
H2…H2	148.8	> 5.0 Å
H5…H5	148.8	> 5.0 Å
H3…H3	148.8	< 4.5 Å (3.5-4.5 Å)
H6…H6	148.8	< 5.0 Å (4.0-5.0 Å)
**1·BF4**		
$H7^{1}$…H2	148.8	> 2.0 Å (2.0-2.5 Å)
$H7^{1}$…H6	148.8	> 2.5 Å (2.5-3.0 Å)
$H7^{1}$…$H7^{1}$	148.8	> 5.0 Å

Table S12 The set of all interatomic distances determined by ssNMR techniques and their comparison with the interatomic distances obtained from SCXRD data.

Spin pair		Interatomic distances *r*, Å	
		ssNMR	SCXRD
**1·BF4**			
F…C2,6	-CH=	4.9 ± 0.4 Å	3.416(6)*, 4.134(7)*, 4.785(7),4.837(6), 5.076(6)
F…C7	-CH2-S-	3.4 ± 0.4 Å	3.287(8), 3.257(6)
F…C8	-N-C-S-	3.8 ± 0.4 Å	3.594(7), 3.692(8), 3.693(7), 3.903(6)
F…C9,10	-CH2-N-	3.1 ± 0.4 Å	3.092(6), 3.104(7), 3.149(7), 3.237(7), 3.246(6), 3.276(6), 3.282(6)
B…B		4.8 ± 0.4 Å	5.073(8)
$H7^1$…H2		> 2.0 Å (2.0-2.5 Å)	
$H7^1$…H6		> 2.5 Å (2.5-3.0 Å)	
$H7^1$…$H7^1$		> 5.0 Å	
**2·BF4**			
F…C1	-C=	4.2 ± 0.4 Å	4.15(3), 4.254(17), 4.26(2), 4.48(2), 4.574(18)
F…C4	-C=	4.6 ± 0.4 Å	4.524(17), 4.79(2), 4.99(2)
F…C7	-CH2-S-	3.2 ± 0.4 Å	3.39(2), 3.45(2)
F…C8	-N-C-S-	3.3 ± 0.4 Å	3.62(3), 3.63(2)
B…B		5.3 ± 0.4 Å	
		Bimodal distribution	
		5.0 ± 0.3 Å	5.11(4), 4.87(3)
		5.5 ± 0.3 Å	5.61(2), 5.607(19)
H2…H2		> 5.0 Å	
H3…H3		< 4.5 Å (3.5-4.5 Å)	
H5…H5		> 5.0 Å	
H6…H6		< 5.0 Å (4.0-5.0 Å)	
**3·BF4**			
F…C1	-CH=	4.5 ± 0.5 Å	5.112(3)*
F…C3	-CH=	3.4 ± 0.4 Å	3.388(2)
F…C11	-CH2-S-	3.2 ± 0.4 Å	3.185(3),
F…C12	-N-C-S-	3.4 ± 0.4 Å	3.406(2), 3.409(3), 3.493(3), 3.580(3)
B…B		5.8 ± 0.4 Å	5.801(3), 4.698(4)
		4.5 ± 0.4 Å	

*the closest A...B distance, but does not satisfy the condition.

S5.4. ^{1}H-^{13}C FSLG HETCOR NMR spectra for 3·BF4, 2·BF4 and 1·BF4

Compound **2·BF4**

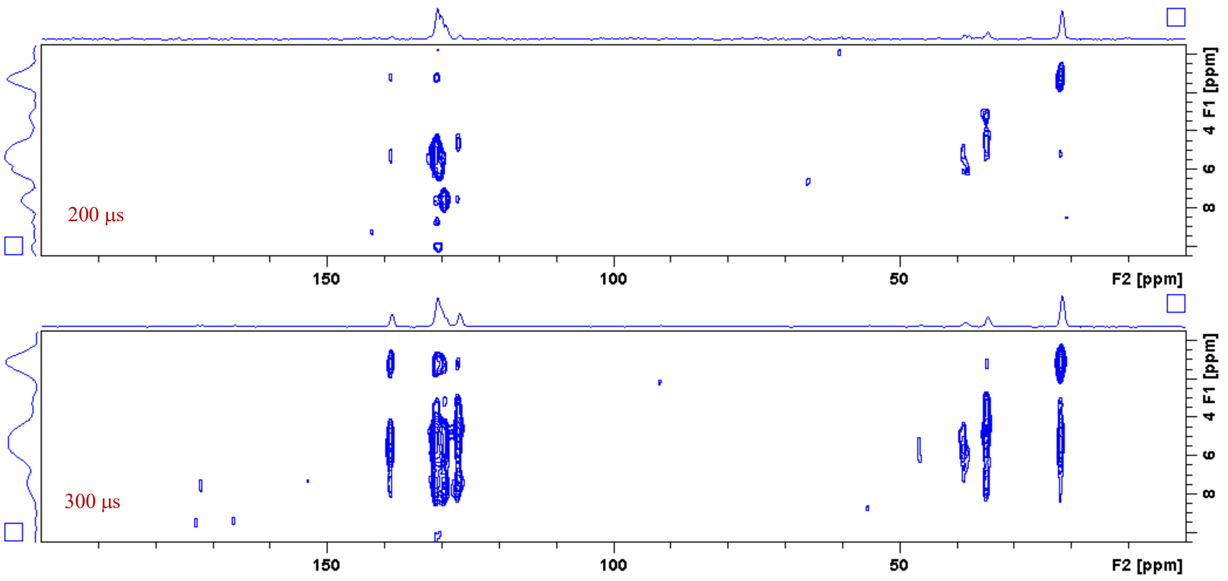

Figure S20 ^{1}H-^{13}C FSLG HETCOR NMR spectra of **2·BF4** measured at different cross-polarization mixing times P15 200 and 300 µs.

Compound **1·BF4**

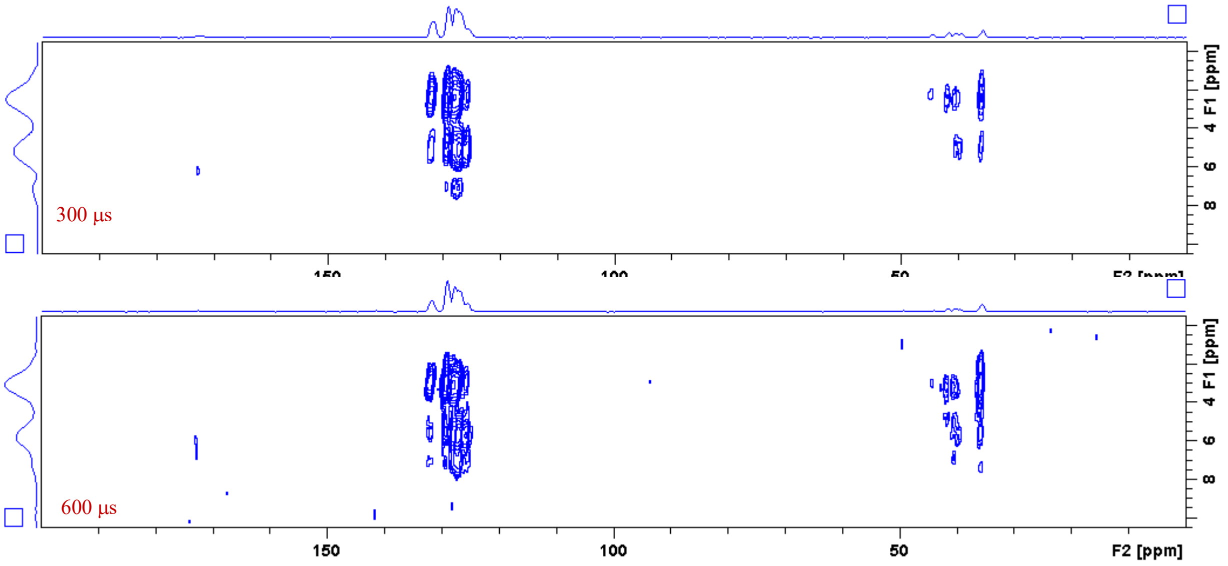

Figure S21 ^{1}H-^{13}C FSLG HETCOR NMR spectra of **1·BF4** measured at different cross-polarization mixing times P15 300 and 600 µs.

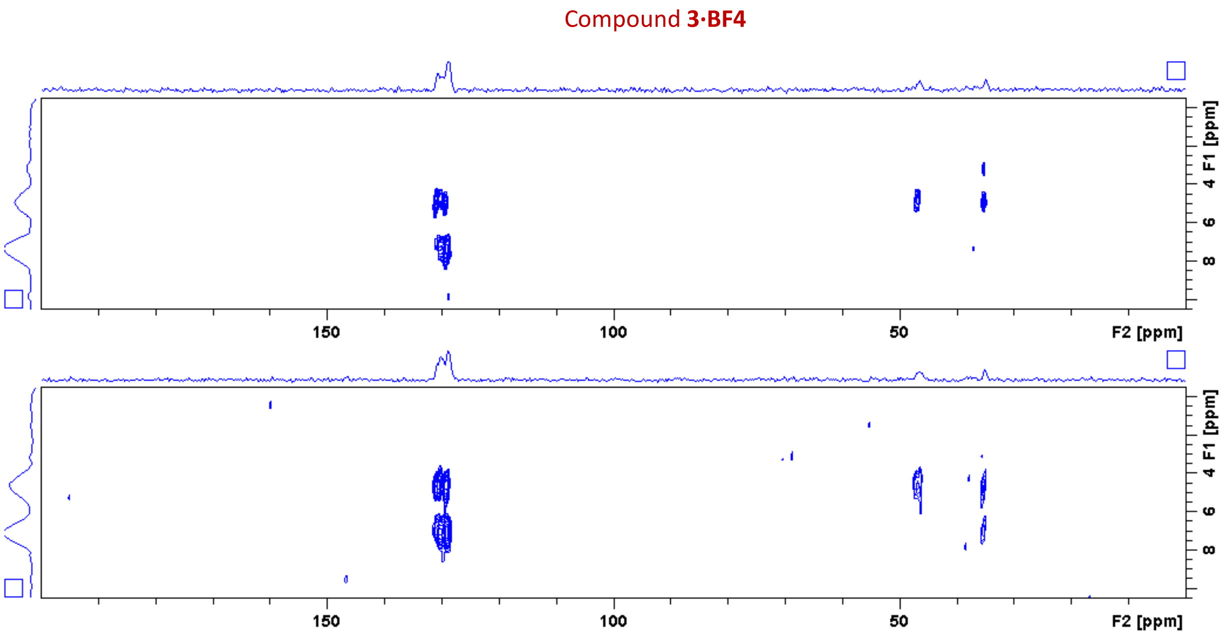

Figure S22 ^{1}H-^{13}C FSLG HETCOR NMR spectra of **3·BF4** measured at different cross-polarization mixing times P15 200 and 300 μs.

It is now generally accepted that the correlation signals in ^{1}H-^{13}C FSLG HETCOR spectra reflecting one-bond polarization transfer of 1.1 Å are developed within the CP mixing time of 70-100 μs, whereas medium-range correlations corresponding to an interatomic ^{1}H...^{13}C distance of about 2.5 Å require CP mixing time of 220-250 μs [20,21]. The evolution of long-range correlation signals involving both direct ^{1}H-^{13}C as well as relayed ^{1}H-^{1}H-^{13}C polarizations transfer then requires mixing times up to ca. 600-800 μs.

S6. Results of XRPD

X-ray powder diffraction data were collected using the Bragg-Brentano focusing configuration on the powder diffractometer Empyrean of PANalytical ($\lambda_{Cu,K\alpha}$ = 1.54184 Å) that was equipped with fixed divergent slit and PIXcel3D detector. The sample was gently ground and placed in the flat-sample holder. The 30-minute measurements were made from 4 to 40 °2Theta with 0.013° step size.

The measured powder patterns were fitted in Jana2020 using the fixed structural models. The unit cell parameters were refined along with the profile parameters, global ADP parameter and scale factor. Although the structures were solved by SCXRD at different temperatures than the measured powder data, the resulting agreement was acceptable and further refinement, such as a rigid body refinement, was not required to confirm the purity of samples.

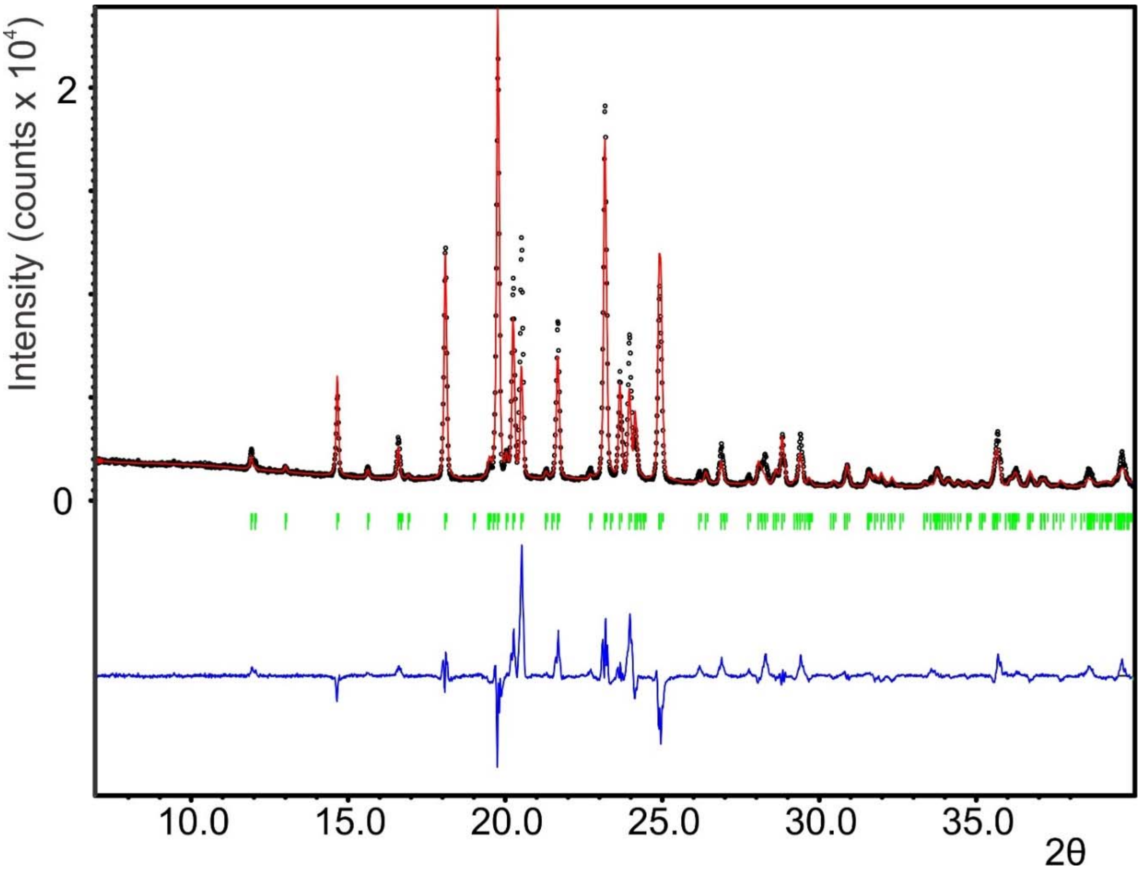

Figure S23 Whole pattern profile fit of the **1·BF4** with fixed model. Only unit cell, zero shift, global ADP parameter and profile were refined. The red line is the calculated profile, black dots are measured data, the blue line means the difference curve and green bars are Bragg positions.

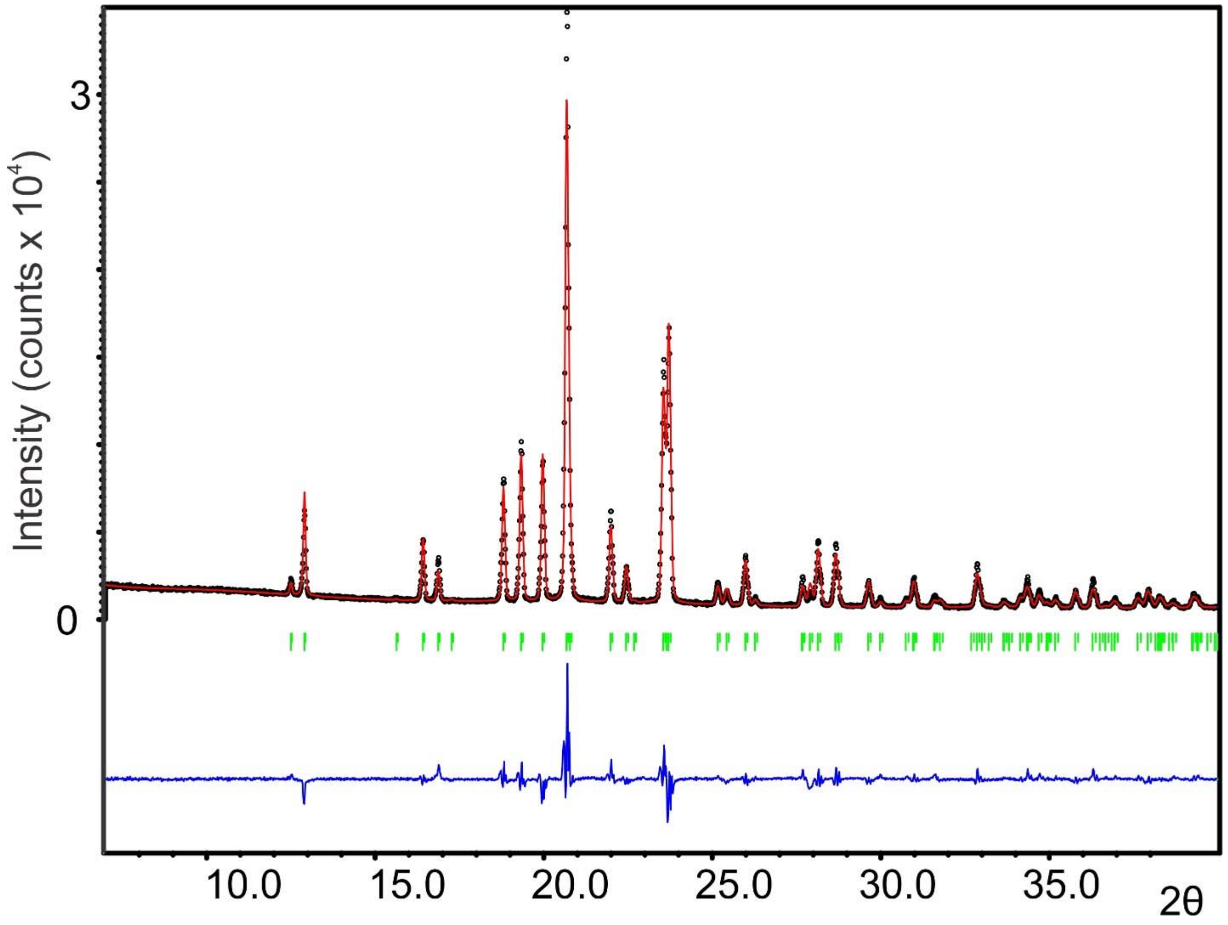

Figure S24 Whole pattern profile fit of the **2·BF4** with fixed model. Only unit cell, zero shift, global ADP parameter and profile were refined. The red line is the calculated profile, black dots are measured data, the blue line means the difference curve and green bars are Bragg positions.

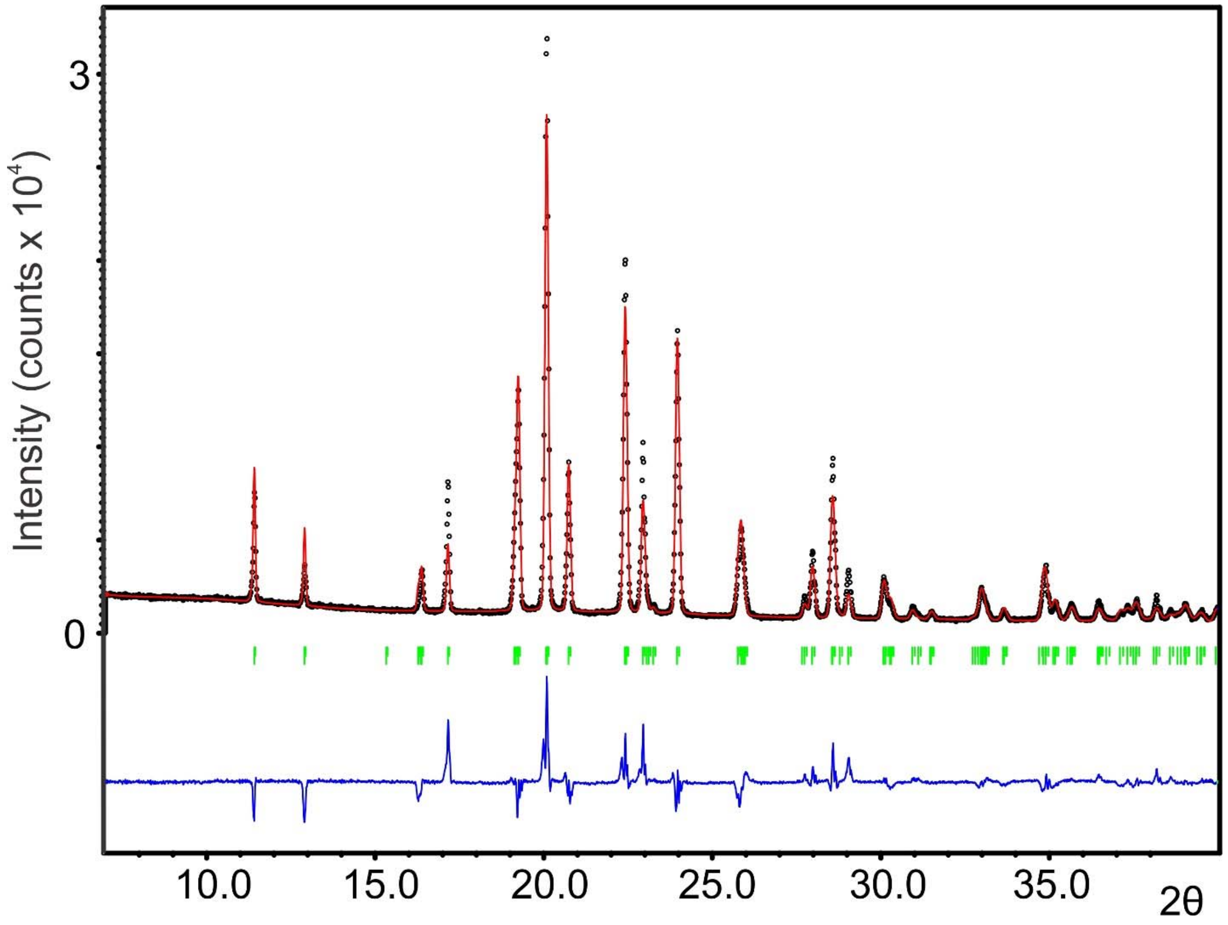

Figure S25 Whole pattern profile fit of the **3·BF4** with fixed model. Only unit cell, zero shift, global ADP parameter and profile were refined. The red line is the calculated profile, black dots are measured data, the blue line means the difference curve and green bars are Bragg positions.

References

1 E. Salager, R. S. Stein, S. Steuernagel, A. Lesage, B. Elena and L. Emsley, *Chem. Phys. Lett.*, 2009, **469**, 336–341.

2 S. P. Brown, A. Lesage, B. Elena and L. Emsley, *J. Am. Chem. Soc.*, 2004, **126**, 13230–13231.

3 M. Hohwy, C. M. Rienstra, C. P. Jaroniec and R. G. Griffin, *J. Chem. Phys.*, 1999, **110**, 7983–7992.

4 B.-J. Van Rossum, H. Förster and H. J. M. De Groot, *J. Magn. Reson.*, 1997, **124**, 516–519.

5 I. Schnell, B. Langer, S. H. M. Söntjens, M. H. P. Van Genderen, R. P. Sijbesma and H. W. Spiess, *J. Magn. Reson.*, 2001, **150**, 57–70.

6 S. P. Brown and H. W. Spiess, *Chem. Rev.*, 2001, **101**, 4125–4156.

7 J. Brus, J. Czernek, M. Urbanova, L. Kobera and A. Jegorov, *Phys. Chem. Chem. Phys.*, 2017, **19**, 487–495.

8 M. Husak, A. Jegorov, J. Rohlicek, A. Fitch, J. Czernek, L. Kobera and J. Brus, *Cryst. GROWTH Des.*, 2018, **18**, 3616–3625.

9 M. Edén, D. Zhou and J. Yu, *Chem. Phys. Lett.*, 2006, **431**, 397–403.

10 Q. Wang, B. Hu, O. Lafon, J. Trébosc, F. Deng and J. P. Amoureux, *J. Magn. Reson.*, 2009, **200**, 251–260.

11 B. Langer, I. Schnell, H. W. Spiess and A.-R. Grimmer, *J. Magn. Reson.*, 1999, **138**, 182–186.

12 J. Brus, *Solid State Nucl. Magn. Reson.*, 2000, **16**, 151–160.

13 CrysAlis PRO Rigaku Oxford Diffraction Ltd, Yarnton, Oxfordshire, England 2020.

14 L. Palatinus and G. Chapuis, *J. Appl. Crystallogr.*, 2007, **40**, 786–790.

15 V. Petříček, L. Palatinus, J. Plášil and M. Dušek, *Z. Für Krist. - Cryst. Mater.*, 2023, **238**, 271–282.

16 P. W. Betteridge, J. R. Carruthers, R. I. Cooper, K. Prout and D. J. Watkin, *J. Appl. Crystallogr.*, 2003, **36**, 1487–1487.

17 J. Rohlíček and M. Hušák, *J. Appl. Crystallogr.*, 2007, **40**, 600–601.

18 K. Brandenburg, DIAMOND Crystal Impact GbR, Bonn, Germany 1999.

19 C. F. Macrae, I. Sovago, S. J. Cottrell, P. T. A. Galek, P. McCabe, E. Pidcock, M. Platings, G. P. Shields, J. S. Stevens, M. Towler and P. A. Wood, *J. Appl. Crystallogr.*, 2020, **53**, 226–235.

20 B.-J. Van Rossum, H. Förster and H. J. M. De Groot, *J. Magn. Reson.*, 1997, **124**, 516–519.

21 J. Brus and A. Jegorov, *J. Phys. Chem. A*, 2004, **108**, 3955–3964.
